# Edible Pneumatic Battery for Sustained and Repeated Robot Actuation

**DOI:** 10.1002/advs.202509350

**Published:** 2025-10-24

**Authors:** Bokeon Kwak, Shuhang Zhang, Alexander Keller, Qiukai Qi, Jonathan Rossiter, Dario Floreano

**Affiliations:** ^1^ Laboratory of Intelligent Systems, School of Engineering Ecole Polytechnique Fédérale de Lausanne (EPFL) Lausanne 1015 Switzerland; ^2^ School of Engineering Mathematics and Technology University of Bristol Bristol BS16 1QY UK

**Keywords:** biodegradable robots, edible robots, edible energy production and storage, edible battery, edible valve

## Abstract

Energy production and storage represent challenges for biodegradable and edible technologies. Here, this study describes an edible energy storage and valve system designed to power pneumatically driven edible robots. The edible pneumatic battery exploits the acid‐base neutralization reaction of food‐grade reactants: under gravity, citric acid mixes with sodium bicarbonate powder to produce a steady release of carbon dioxide (CO_2_) gas. The generated gas pressure causes deformation of a connected edible pneumatic actuator. When the gas pressure reaches a threshold, an edible valve automatically releases the pressurized gas, which lets the actuator return to its resting state. The entire system, whose characteristics are consistent with model estimates, is fully edible and enables self‐sustained and repetitive bending motion of the edible actuator. This design is scalable in terms of sizes (30–50 mm diameter), operation time (20–650 s), and CO_2_ gas generation rate (0.1–1.4 × 10^−3 ^mol s^−1^). Additionally, the actuator's motion can be programmed by modifying the orifice size or the fluidic resistance between the energy source, actuator, and valve. The system is validated by fabricating a fully edible system, and its application is showcased as a foot‐pressed triggered edible actuator that mimics prey behavior to attract predators.

## Graphical abstract

This work presents an edible energy source and valve system to power soft, pneumatically driven edible robots. A chemical reaction between sodium bicarbonate and citric acid generates carbon dioxide gas, and a pressure‐triggered edible valve enables self‐repetitive motion of the edible actuator. The system is scalable, programmable, and demonstrated through a foot‐triggered actuator that potentially mimics prey to attract wildlife.

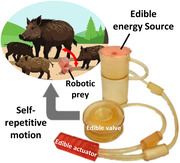

## 1 Introduction

Fluidic actuation is widely used in soft and biodegradable robots where the energy sources typically are positive or negative pressure motor‐driven pumps^[^ ^1^, ^2^, ^3^, ^4^, ^5^, ^6^, ^7^ ^]^ or pressurized gas canisters,^[^ ^8^, ^9^ ^]^ the latter exhibiting limited operation time.^[^ ^10^ ^]^ However, all these pressure sources are not biodegradable because they rely on electromagnetic components and electrochemical batteries (e.g., lithium polymer), which produce environmental waste and are toxic.

Gas‐generating chemical reactions have been extensively explored as alternative pneumatic energy sources for soft and biodegradable robots,^[^ ^11^, ^12^, ^13^, ^14^, ^15^, ^16^, ^17^, ^18^, ^19^, ^20^, ^21^, ^34^ ^]^ due to their portability, sustainability, and high energy density.^[^ ^22^ ^]^ Hydrogen peroxide (H_2_O_2_) is a commonly used chemical for powering pneumatic robots,^[^ ^11^, ^12^, ^13^ ^]^ as it naturally decomposes into water (H_2_O) and oxygen (O_2_) gas, with the decomposition process further accelerated by catalysts (e.g., platinum). However, higher concentrations of H_2_O_2_ are corrosive and must be handled with caution.^[^ ^22^, ^23^ ^]^ The generation of hydrogen (H_2_) gas through the breakdown of an aluminum–gallium–indium (Al–Ga–In) alloy in a potassium hydroxide (KOH) solution has also been studied for actuating soft robots.^[^ ^21^ ^]^ However, this method is unsuitable for biodegradable or robotic applications due to the toxicity of the Al–Ga–In alloy.

Gas‐generating biological and acid‐base reactions have been explored as alternative methods by considering their safety and biocompatibility. Carbon dioxide (CO_2_) generation through the hydrolysis of urea by means of urease enzyme^[^ ^18^ ^]^ and of yeast fermentation^[^ ^19^ ^]^ have both been investigated to power soft actuators. However, these methods exhibit slow actuation speeds, with target pressures typically reached after 3 to 5 min. Faster actuation can be achieved by mixing citric acid (naturally found in citrus fruits) with sodium bicarbonate (baking soda), which has been shown to power a pneumatic actuator up to 1 MPa,^[^ ^14^ ^]^ a gelatin actuator,^[^ ^15^ ^]^ and a fully edible robotic miniature boat.^[^ ^34^ ^]^ However, the system in^[^ ^14^ ^]^ was bulky, and many components (e.g., flask, tube, and valve) were not biodegradable/edible. While all materials in^[^ ^15^ ^]^ are edible, the pressure‐generating components are encapsulated within the actuator, thus making it difficult to use a single power generator for multiple actuator types. The water‐triggered energy source used in^[^ ^34^ ^]^ lacks a gas‐releasing valve, and this cannot sustain repeated operation of an actuator.

A gas‐generating reaction provides only positive pressure, and thus it alone cannot achieve repetitive motion of a soft actuator. Some previous studies have coupled gas‐consuming reactions (which provide negative pressure) with gas‐generating reactions to enable pressure regulation of soft robotic devices.^[^ ^16^, ^17^, ^20^ ^]^ However, they either required manual regulation of a reactant during the chemical reaction,^[^ ^16^ ^]^ an inedible thermoelectric material,^[^ ^17^ ^]^ or large electric power sources that are not biodegradable or edible.^[^ ^17^, ^20^ ^]^ Previous soft robots powered by chemical reactions could display self‐oscillating actuation by means of an oscillatory microfluidic circuit,^[^ ^11^ ^]^ or an elastomeric deflector that self‐regulates the chemical supplies.^[^ ^12^ ^]^ However, none of the above oscillation mechanisms are edible/biodegradable and require substantial modification to achieve such transition. Here, in contrast, we develop an edible pneumatic battery specifically from edible materials, through an integrated structure, actuation, and materials design process.

**Figure 1:**
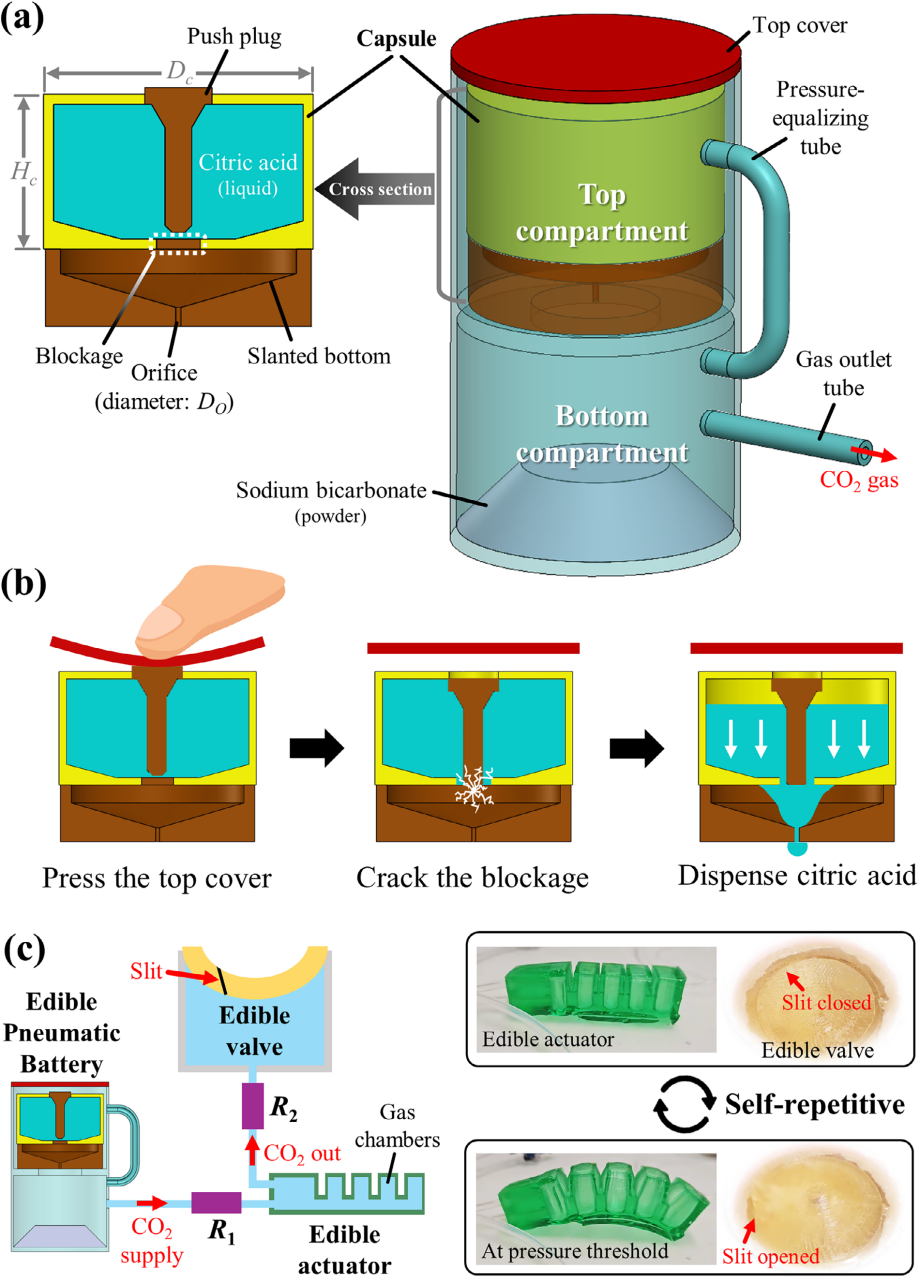
Schematic representation of the proposed edible battery‐actuator system to generate rhythmic and sustained gas generation and release. a) Edible pneumatic battery and cross‐section of the edible capsule containing liquid citric acid, which is stored in the top compartment. b) Pressure applied to the top of the battery cover cracks the blockage, and citric acid starts flowing out via an orifice. It mixes with sodium bicarbonate powder underneath, generating CO_2_ gas (Video S1, Supporting Information). c) the gas produced by the pneumatic battery flows into a soft edible actuator and out into an edible valve with a slit which opens and releases gas when the pressure exceeds a certain threshold, thus depressurizing the actuator. *R*_1_ and *R*_2_ are two fluidic resistors to regulate the pressure levels of the edible actuator and valve (see also Figure 4f).

The edible pneumatic battery described here relies on the chemical reaction of sodium bicarbonate and citric acid, yielding an energy source that is, safe to eat, delivers fast actuation, is low cost, and has zero environmental impact. To deliver a regulated and sustained source of gas, liquid citric acid is dispensed onto sodium bicarbonate powder through an internal orifice (**Figure** **1**a,b). The edible pneumatic battery is a standalone device that can be used to drive a range of biodegradable or edible pneumatic actuators. The periodic and repeated powering of the actuator is enabled by an edible tubing and valve system that regulates the intake and release of gas within the actuator. The entire battery‐actuator system can generate rhythmic and sustained gas‐driven actuation (Figure 1c). We validate the edible pneumatic battery and valve system in a proof‐of‐concept prey robot that could be used to deliver nutrition or vaccines to reclusive terrestrial animals. The robot is buried underground and reveals itself with repetitive motion when the pneumatic battery is activated by the animal. Note that we intentionally do not specify the intended consumers, as this work is not aimed at immediate real‐world application. In the future, factors such as taste, nutritional profile, desired range and duration of motion, and the level of food hygiene during production should be tailored to specific consumers and applications.

## 2 Results

### 2.1 Pneumatic Battery

The two edible reagents, citric acid and sodium bicarbonate, are stored in two separate compartments of the battery. The top compartment contains the capsule with liquid citric acid, and the bottom compartment contains the powdered sodium bicarbonate (see Figure 1a). To enable prolonged and controlled mixing of the reagents, a small orifice is placed beneath the edible capsule. The top and bottom compartments are connected by a pressure‐equalizing tube, inspired by a dropping funnel,^[^ ^16^ ^]^ which maintains a zero pressure difference across the orifice (otherwise a pressure difference would evolve and affect acid flow and gas generation rate). When the top cover is manually pressed, a plug breaks a blockage and citric acid starts dripping from the top compartment into the bottom compartment (see Figure 1b; Video S1, Supporting Information). The edible pneumatic battery is assembled by bonding molded edible components made of gelatin with varying amounts of plasticizers and edible waxes, (see Figure S1, Supporting Information, for the fabrication process). The discharge of citric acid (C_6_H_8_O_7_) onto sodium bicarbonate (NaHCO_3_) is driven by gravity, which generates CO_2_ gas according to:

(1) $$C_{6}H_{8}O_{7}+3\mathrm{NaHC}O_{3}\to Na_{3}C_{6}H_{5}O_{7}+3H_{2}O+3CO_{2}$$

The reaction between one mole of citric acid and three moles of sodium bicarbonate produces one mole of sodium citrate (Na_3_C_6_H_5_O_7_), three moles of water (H_2_O), and three moles of CO_2_ gas as by‐products.^[^ ^22^ ^]^ Sodium citrate is an edible material commonly used as a food additive to adjust hardness, texture, and taste.^[^ ^24^ ^]^ The CO_2_ gas serves as the energy source for bending an edible pneumatic actuator whose internal pressure is automatically regulated by an edible valve with a slit (see Figure 1c). The slit opens when the pressure exceeds its threshold, releasing CO_2_ gas, and then quickly closes (see Video S2, Supporting Information). This cycle of opening and closing the slit repeats while the pneumatic battery continues to produce gas. More details about the edible valve are given below in Section 2.3.

Our system (including the pneumatic battery, actuator, and valve) is technically edible, as it is made solely from authorized food additives with designated E numbers: gelatin (E441), glycerol (E422), beeswax (E901), carnauba wax (E903), sodium bicarbonate (E500), and citric acid (E330). All these materials pose no known environmental risks. However, any potential risks to consumers should be carefully assessed when specific applications for our system are considered in the future. For instance, if the system is designed to deliver nutrition or vaccines (by infusing them into the edible actuator) to elusive wild animals, long‐term toxicological studies involving live animals are necessary. Even if our system is deployed but not consumed, it can still biodegrade due to the inherent biodegradability of the food materials used. As a result, our system is environmentally friendly and leaves little to no waste behind.

### 2.2 Characterization

**Figure 2:**
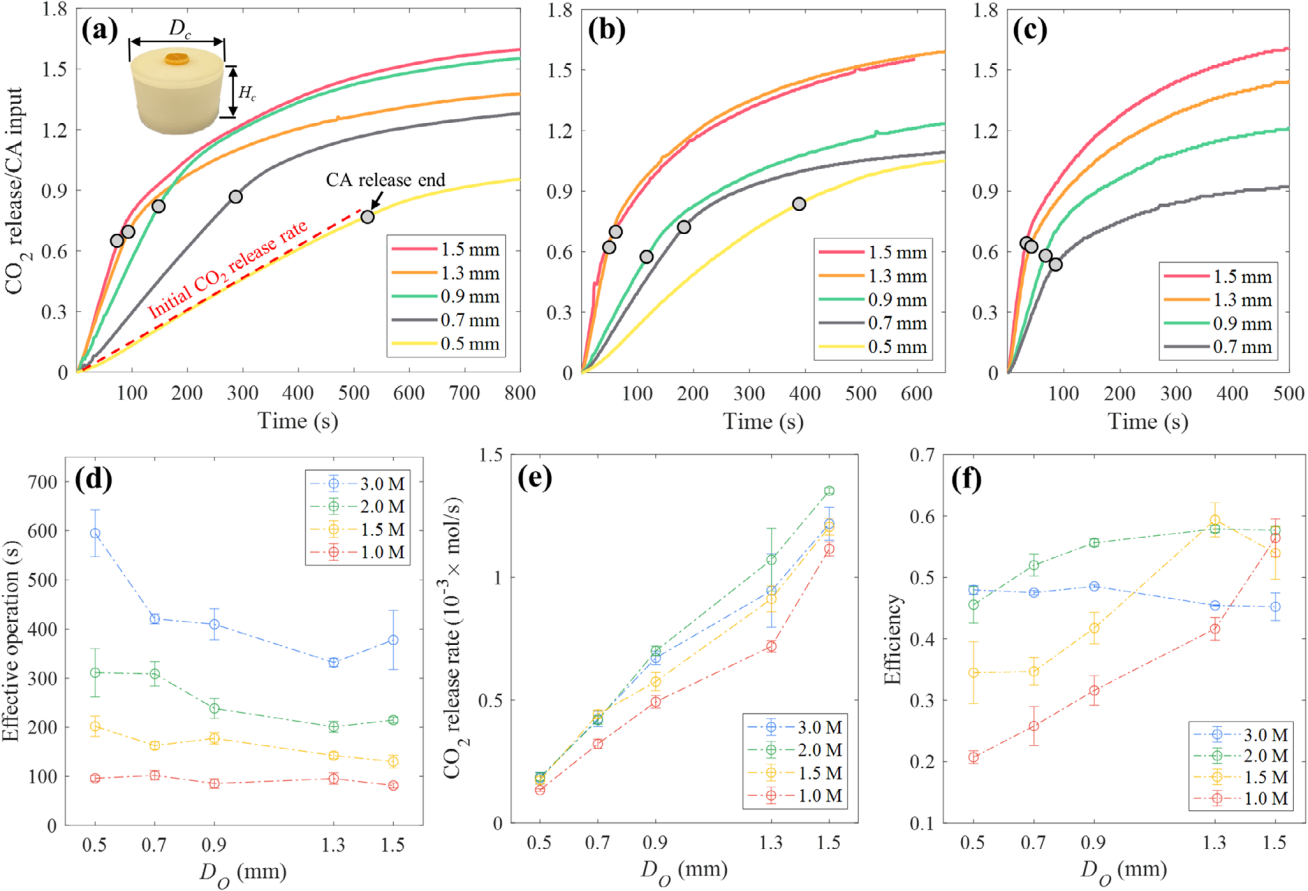
Characterization of the edible pneumatic battery. a–c) CO_2_ released normalized with the molar mass of citric acid (CA) at a 3.0 m concentration (i.e., 3 moles of CA in 1 L of water) during the chemical reaction with five different diameters of orifice (*D*_O_). The grey markers indicate the moment when the CA release is ended ((a) Large capsule, (b) Medium capsule, (c) Small capsule). The CO_2_ release curves for other CA concentrations (1.0, 1.5, and 2.0 m) exhibited similar trends (see Figure S3, Supporting Information). d) The effective operation time (i.e., the elapsed time until the CO_2_ release rate drops below 10% of the maximum flow rate of a commercial microcompressor^[^ ^10^ ^]^) of the pneumatic battery as a function of CA concentration and orifice diameter (*D*_O_). e) CO_2_ release rate at the beginning of each reaction (e.g., red dashed line in Figure 2a when *D*_O_ = 0.5) for the large capsule. CO_2_ release rate is reduced in medium and small capsules (see Figure S6, Supporting Information), as a large capsule creates a higher hydrostatic pressure. f) Comparison of efficiency from a large capsule; the total mass of CO_2_ released during effective operation divided by the total possible CO_2_ release if all CA molecules from a capsule were to fully react with sodium bicarbonate. Each case was tested three times (n = 3) in (d–f).

Comprehensive characterizations were taken to measure CO_2_ gas release rate, total gas output, and operating time of the pneumatic battery for different concentrations of citric acid (1.0, 1.5, 2.0, and 3.0 m), orifice diameters (*D*_o_ = 0.5, 0.7, 0.9, 1.3, 1.5 mm), and size of the capsules with 1.5 mm of wall thickness (see Figure 1a or the inlet of Figure 2a: small; *D*_c_ = 30 mm, *H*_c_ = 16.5 mm; medium; *D*_c_ = 40 mm, *H*_c_ = 22 mm; large; *D*_c_ = 50 mm, and *H*_c_ = 27.5 mm) fully containing citric acid. Note that the ratio *D*_c_ /*H*_c_ is all the same for each capsule size. Each capsule also contains the same volume of citric acid (small: 7.6 mL, medium: 16.0 mL, and large: 34.6 mL) at different concentrations, and the mass of sodium bicarbonate is adjusted to perfectly balance the reaction. The total mass of citric acid (CA) and sodium bicarbonate decreases over time due to the gas‐producing chemical reaction, which was measured with a scale (Figure S2, Supporting Information). Additionally, each combination of modeled dimensions was prototyped and experimentally measured with the exception of a small capsule with *D*_O_ = 0.5 mm, which could not release CA caused by the increased effect of capillary pressure over hydrostatic pressure (see Note S1 and Tables S1 and S2, Supporting Information).

The orifice (see Figure 1a) allowed CA to gradually mix with sodium bicarbonate, resulting in a constant CO_2_ release rate at the beginning of the reaction (see **Figure** **2**a–c), which continued until the release of CA ended (highlighted with grey markers in Figure 2a–c). The time required to release CA from a large capsule is reduced from 499 to 74 s by increasing *D_O_* from 0.5 to 1.5 mm (Figure 2a). CA can be released up to 15% faster when CA concentration was reduced from 3.0 to 1.0 m (Figure S4, Supporting Information), due to the reduced kinematic viscosity of CA in lower concentration (see Note S1, Supporting Information).^[^ ^25^, ^26^, ^27^, ^28^ ^]^ The same trend is observed in medium and small capsules (Figure 2b,c; Figures S3 and S4, Supporting Information).

Some CO_2_ was still produced after the CA release stopped, but at a lower rate (Figure 2a–c), which corresponds to low actuation frequency by ideal gas law.^[^ ^22^ ^]^ To avoid counting a low CO_2_ release rate as the operation time of an edible battery, we define effective operation as the elapsed time from the start of the chemical reaction to when the CO_2_ release rate drops below 10% of the maximum flow rate of a commercial microcompressor, 1.2 × 10^−4^ mol s^−1^.^[^ ^10^ ^]^ Overall, prolonged operation of the pneumatic battery can be achieved with a higher CA concentration, smaller *D*_O_, and larger capsule size (Figure 2d; Figure S5, Supporting Information). For instance, a large capsule with *D*_O_ = 0.5 mm has effective operations of 96, 202, 311, and 595 s when its CA concentration is 1.0, 1.5, 2.0, and 3.0 m, respectively (Figure 2d).

Meanwhile, using a larger *D*_O_ (Figure 2e) and larger capsule size (Figure S6, Supporting Information) is beneficial for a fast CO_2_ release rate (i.e., fast actuation), as CA is more rapidly released from the capsule owing to the increased hydrostatic pressure.^[^ ^25^ ^]^ In the case of 2.0 m, the CO_2_ release rate is enhanced from 0.19 × 10^−3^ to 1.35 × 10^−3^ mol s^−1^ as *D*_O_ increases from 0.5 to 1.5 mm (Figure 2e). Note that 2.0 m generally exhibits a faster CO_2_ release rate than 3.0 m in all three capsule sizes (Figure 2e; Figure S6, Supporting Information), suggesting that the water content in 3.0 m CA is insufficient to mix CA molecules with dry sodium bicarbonate powder. This attribute 2.0 m CA to generally exhibit better gas‐release efficiency than 3.0 m CA (Figure 2f). Similar to the CO_2_ release tendency in Figure 2e, larger *D*_O_ (except 3.0 m) and larger capsule size lead to better efficiency (Figure 2f; Figure S7, Supporting Information), as this combination leaves less CA residual inside a capsule (Figure S8, Supporting Information).

### 2.3 Edible Valve

**Figure 3:**
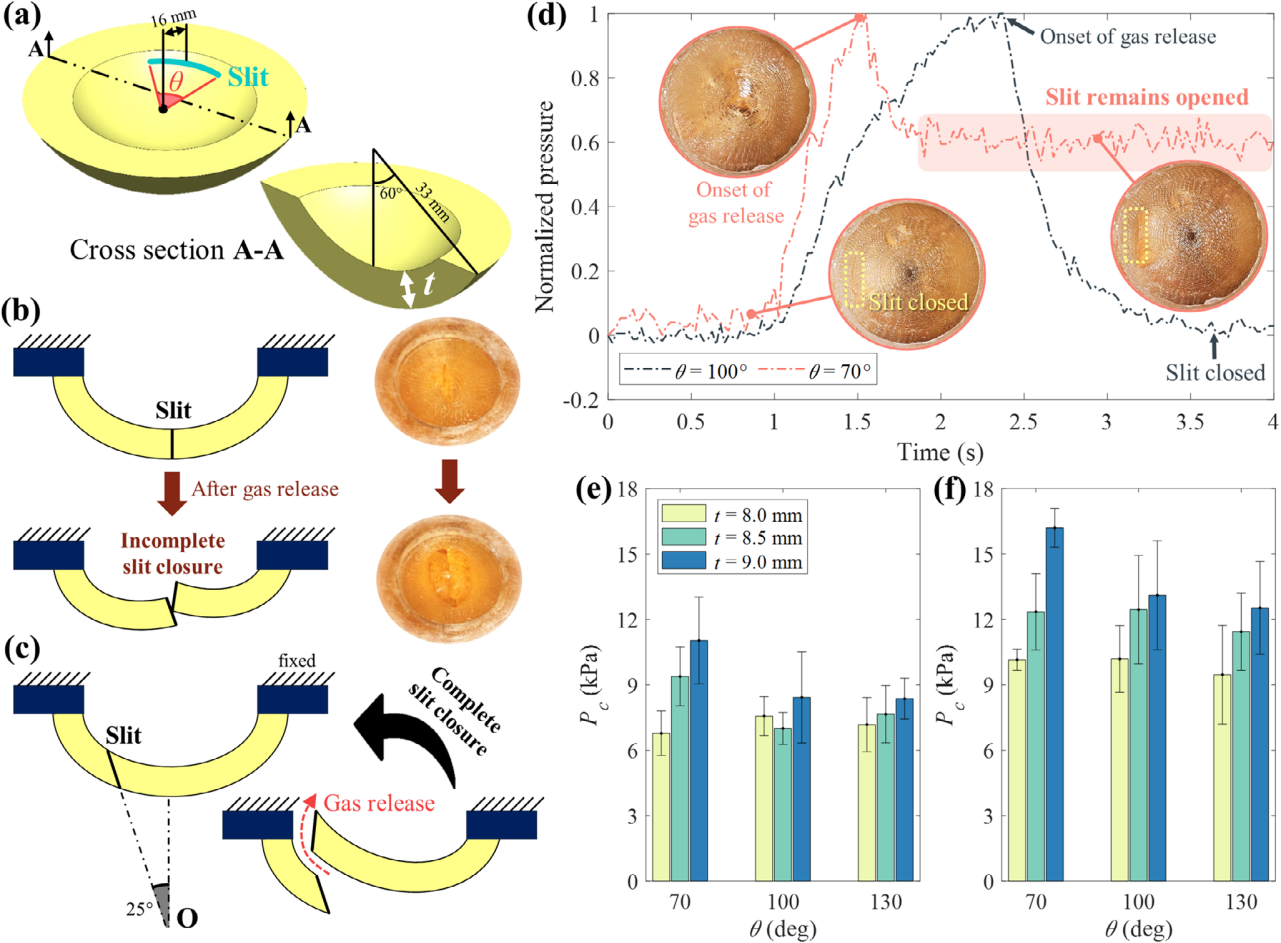
Design and characterization of an edible valve. a) An edible valve with a shell thickness of *t* has a *θ*‐degree circumferential slit at radius 16 mm. b) A non‐tilted slit located at the center is often incompletely closed after gas release; thus this design is not considered in the rest of the paper. c) the slit is located off the central axis and tilted toward the center O at 25° to facilitate complete slit closure after releasing pressurized gas. d) the pressure profile of the round slit valve upon pressurized gas input with two different slit angles; a small slit angle (*θ* = 70°) caused the valve to remain partially opened after gas release, unlike *θ* = 100°; the slit remain closed after gas release. e,f) the critical pressure *P*_C_ of the edible valve at the onset of gas release in two different gelatin, water, and glycerol mixture ratios (e: 1:2.5:2.5, f: 1:2:2 in mass); Each case was tested five times (n = 5).

Several types of soft valves for autonomous pressure regulation have been described, such as an edible linear slit valve,^[^ ^15^ ^]^ an inedible spherical membrane with three slits converging at its apex,^[^ ^3^ ^]^ and inedible spherical metacap with an aperture and arrays of ribs.^[^ ^9^ ^]^ Instead of replicating the inedible valves from^[^ ^3^, ^9^ ^]^ using edible materials, we modified the edible valve design from^[^ ^15^ ^]^ to better suit an edible pneumatic battery. Although the edible valve in^[^ ^15^ ^]^ demonstrated repetitive pressure release at the scale of 1 Pa, this range is too low for edible pneumatic actuators that typically require pressure levels of several kPa.^[^ ^31^ ^]^ Here, we used a valve that leverages snap‐buckling to open and then recover its original shape upon closing. The valve is designed as a monostable (i.e., buckled state instantly snaps back to its initial state after release^[^ ^29^ ^]^), shallow circular shell with a round slit is cut on the shell off the central axis (**Figure** **3**a). The valve remains closed at resting state. As pressure increases, the shell gradually deforms upward while the valve is still closed. The valve opens and releases gas once the pressure exceeds a certain threshold. As soon as the pressure drops, the shell snaps back to its initial shape, and this cycle is repeated (Video S2, Supporting Information). Gelatin was selected as the edible material for prototyping the valve due to its proven ability to develop an edible monostable shell.^[^ ^29^ ^]^

Both the position and orientation of the slit are important for the valve's operation. If the slit is at the center of the shell, the valve does not fully close after gas release and is unsuitable for repetitive gas release (Figure 3b; Figure S10, Supporting Information). In contrast, positioning the slit 16 mm off‐center (Figure 3a) and orienting it at 25° toward point O (Figure 3c) significantly improves valve closure following gas release. With the position (16 mm off‐center) and orientation (25° toward point O) fixed, the effect of the slit angle *θ* (Figure 3a) is investigated in Figure 3d by applying pressure with a syringe (Figure S11, Supporting Information). When *θ* = 100°, the slit fully closed after gas release, and a similar result was observed at *θ* = 130°. In contrast, at *θ* = 70°, the pressure failed to return to zero due to incomplete gas release, and the slit remained partially open (Figure 3d). This occurred because the narrower slit could not release the pressurized gas quickly enough.

To quantify a critical pressure (*P*_c_) at which the edible valve opens, a pressurized air was supplied by a syringe, while the valve was fixed onto a container (see Figure S11, Supporting Information). Three different shell thickness (*t* in Figure 3a) and three *θ* (70°, 100°, and 130°) were considered. These valve designs were manufactured using two types of edible gelatin mixtures (gelatin:glycerol:water = 1:2:2 and 1:2.5:2.5 by mass) to achieve a similar level of softness as that of a previously developed edible pneumatic actuator.^[^ ^7^, ^31^ ^]^ Please refer to Note S3 (Supporting Information) for a more detailed explanation of the rationale behind using gelatin as the main material.^[^ ^36^, ^37^, ^38^, ^39^, ^40^ ^]^ *P*_c_ was increased proportionally to shell thickness *t* because the shell's bending stiffness is proportional to *t^3^*.^[^ ^30^ ^]^ Decreasing the amount of glycerol and water in the gelatin mixture (from 1:2.5:2.5 to 1:2:2) increased *P*_c_ as shown in Figure 3f, due to the increased material stiffness of gelatin hydrogel.^[^ ^29^ ^]^ *P*_C_ values in Figure 3e,f are comparable to the operating pressure range of edible pneumatic actuators,^[^ ^7^, ^31^ ^]^ thereby suitable for self‐sustained actuation. In the end, an edible valve with *t* = 9 mm, *θ* = 100°, and 1:2:2 gelatin mixture is chosen for the remaining experiments, due to its high *P*_c_, which allows for a greater range of motion in the edible actuator.

### 2.4 Self‐Sustained and Repeated Actuation

**Figure 4:**
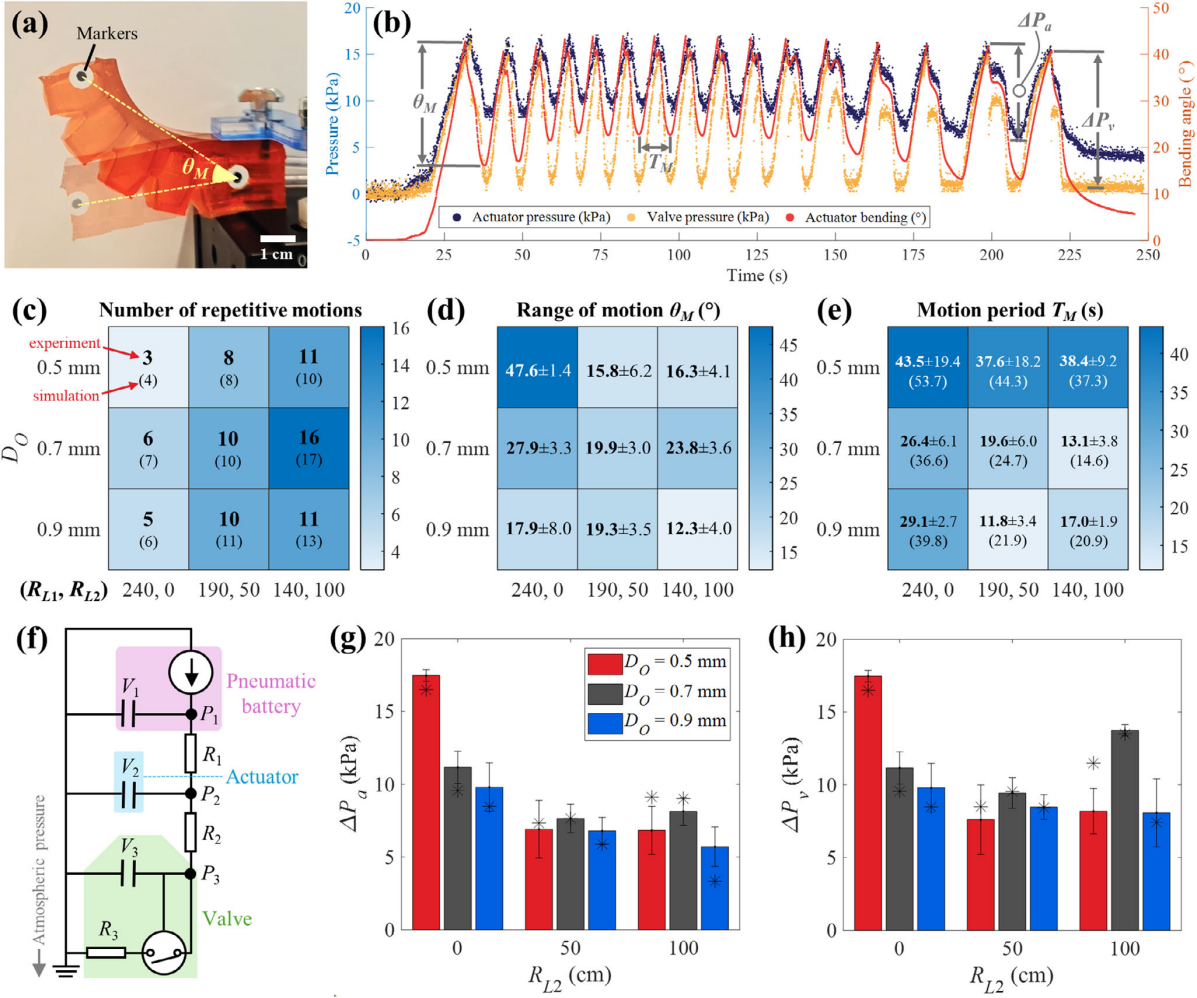
Self‐sustained repeated motion of an edible actuator driven by the edible pneumatic battery (large capsule, 34.6 mL of 2.0 m citric acid solution) and the edible valve. a) Two markers are attached to the edible actuator to measure its motion (*θ*_M_: range of motion, scale bar; 1 cm). b) The evolution of pressure and bending angle when the length of *R*_1_ and *R*_2_ (see Figure 1c) are 140 and 100 cm, respectively (pneumatic battery capsule: large, *D*_O_: 0.7 mm, valve type; *t* = 9 mm, *θ* = 100°). c–e) The number of repetitive bending motion, range of motion *θ_M_*, and motion period *T_M_* (see Figure 4b) for different combinations of *D_O_* and the length of *R*_1_ and *R*_2_, (i.e., *R*_L1_ and *R*_L2_; unit: cm) where simulated results are reported in parenthesis in (c,e). Each case was repeated three times, and then the one that exhibited the highest number of repetitive motions is depicted in (c). The number of data points in *θ*_M_, *T*_M_, *ΔP*_a_, and *ΔP*_v_ is the same as the number of repetitive motions. For example, when *D*_O_ = 0.7 mm and *R*_L1_ = 140 cm (therefore, *R*_L2_ = 100 cm), the number of data points to calculate its *θ*_M_, *T*_M_, *ΔP*_a_, and *ΔP*_v_ is 16. The statistical significance of *θ*_M_ with respect to *D*_O_ (*p*‐value <0.001), *R*_L1_ (*p*‐value <0.001), and the interaction between the two factors (*p*‐value <0.001) is analysed by the two‐way ANOVA (Analysis of Variance). Similarly, the statistical significance of *T*_M_ with respect to *D_O_* (*p*‐value <0.001), *R*_L1_ (*p*‐value <0.002), and the interaction between the two factors (*p*‐value: 0.157) is obtained. f) A pneumatic circuit model corresponds to Figure 1c, where *V*_1_, *V*_2_, and *V*_3_ are internal volumes of the pneumatic battery, actuator, and valve, respectively. *P*_i_ (*i* = 1, 2, and 3) is the pressure at the corresponding node. The pneumatic battery is represented as a constant gas‐generating source. In addition, *R*_3_ is the intrinsic resistance of the slit valve. g,h) the comparison of the magnitude of actuator pressure change (*ΔP_a_*) and valve pressure change (*ΔP*_v_) in simulation (with * marker) and experiment, where *R*_L1_ + *R*_L2_ = 240 cm. Here, *ΔP*_a_ serves as an indirect measure of *θ*_M_.^[^ ^31^ ^]^

To characterize the motion of an edible actuator driven by the system shown in Figure 1c, the actuator, valve, and pneumatic battery were fabricated with edible materials, except tube connections, and an exterior container holding an edible capsule, sodium bicarbonate, and edible valve (see Figure S12, Supporting Information) for efficient repeated measurements. Note that these inedible components are later made of edible materials for the validation of the fully edible system (Section 2.5). The motion of the actuator was tracked by two markers shown in **Figure** **4**a, and the number of repetitive bending motions, range of motion (*θ*_M_), motion period (*T*_M_), and pressure change inside the actuator (*ΔP*_a_) and the valve (*ΔP*_v_) were measured simultaneously, as shown in Figure 4.

We studied the programmability of the actuator by adjusting the orifice diameter (*D*_O_) and fluidic resistance *R*_1_ and *R*_2_ (see Figure 1c) in Figure 4c–e. A thin silicone tube with an inner diameter of 1 mm was used for *R*_1_ and *R*_2_, and their tube lengths (i.e., *R*_L1_ and *R*_L2_) were adjusted to change the resistance by maintaining the sum of *R*_L1_ and *R*_L2_ as 240 cm. The large‐size capsule was used in all the tests, and the medium and small capsules exhibited similar tendencies except for shorter repetitions (Figure S13, Supporting Information). Also, *D*_O_ = 1.3 and 1.5 were not considered as they often puncture the edible actuator due to their higher gas pressure output. However, they are still useful for operating bigger or stiffer edible actuators.

When fixed amount of chemicals (citric acid: 34.6 mL, sodium bicarbonate: adjusted to balance the reaction) is supplied to the pneumatic battery, both *θ*_M_ and *T*_M_ are inversely proportional to the number of repetitive motions, since the chemical potential energy stored in the pneumatic battery is the same. As shown in Figure 4c, the number of repetitive motions increases with a higher *R*_2_. A larger *R*_2_ prevents the actuator's gas outlet from being directly exposed to atmospheric pressure when the valve begins releasing gas at the threshold. Thus, the actuator pressure doesn't drop to zero during the repetitive actuation (see black markers in Figure 4b) and quickly repeats the bending motion (Video S3, Supporting Information). The magnitudes of pressure in the actuator and the valve are almost the same when *R*_2_ = 0 (Figure S14, Supporting Information), unlike the case when *R*_2_ ≠ 0. Letting *R*_2_ = 0 sometimes caused the valve to unexpectedly remain open after gas release and caused the bending angle of the actuator to be zero for a prolong time (see Figure S14 and Video S4, Supporting Information).

The repetitive motion can also be programmed by changing *D*_O_. When *D_O_* = 0.5 mm, the corresponding *T*_M_ were longer than 30 s (Figure 4e) due to the relatively low gas release rate compared to *D_O_* = 0.7 mm and *D_O_* = 0.9 mm (Figure 2e). More irregular motion was observed as the *D_O_* is decreased from 0.9 to 0.5 mm (as indicated by the increased standard deviation of *T*_M_ in Figure 4e). We assume this is caused by minute (but unintended) gas leakage through the slit valve during the pressurization phase. As the valve is made of gelatin hydrogel, it shrinks over time.^[^ ^29^ ^]^ Therefore, once the slit is cut, the gap gradually widened (see Figure S15, Supporting Information), and the valve start losing its functionality after ≈6 h. Thus, the edible valve was replaced every 3 h to ensure its good condition during the experiment.

The system illustrated in Figure 1c can be modeled as a pneumatic circuit (Figure 4f). The pneumatic battery is assumed to include a constant gas‐generating source, while the valve is modeled as a switch that closes the circuit when the pressure reaches *P*_C_ (Figure 3f). Additionally, an inherent fluidic resistance of the slit is assumed to consider the minute gas leakage (discussed in the above paragraph) and gas flow restriction when the valve opens. Note that the valve pressure drop in Figure 4b is not instantaneous, which implies a fluidic delay during gas release. The state equations are provided in Note S2 (Supporting Information), which details the pressure changes of the actuator and the valve over time. Overall, the estimations of a number of repetitive motions (Figure 4c), indirect measure of range of motion (Figure 4g), and pressure change of edible valve (Figure 4h) are well matched with the corresponding experiment.

### 2.5 Proof‐of‐Concept Robotic Prey

**Figure 5:**
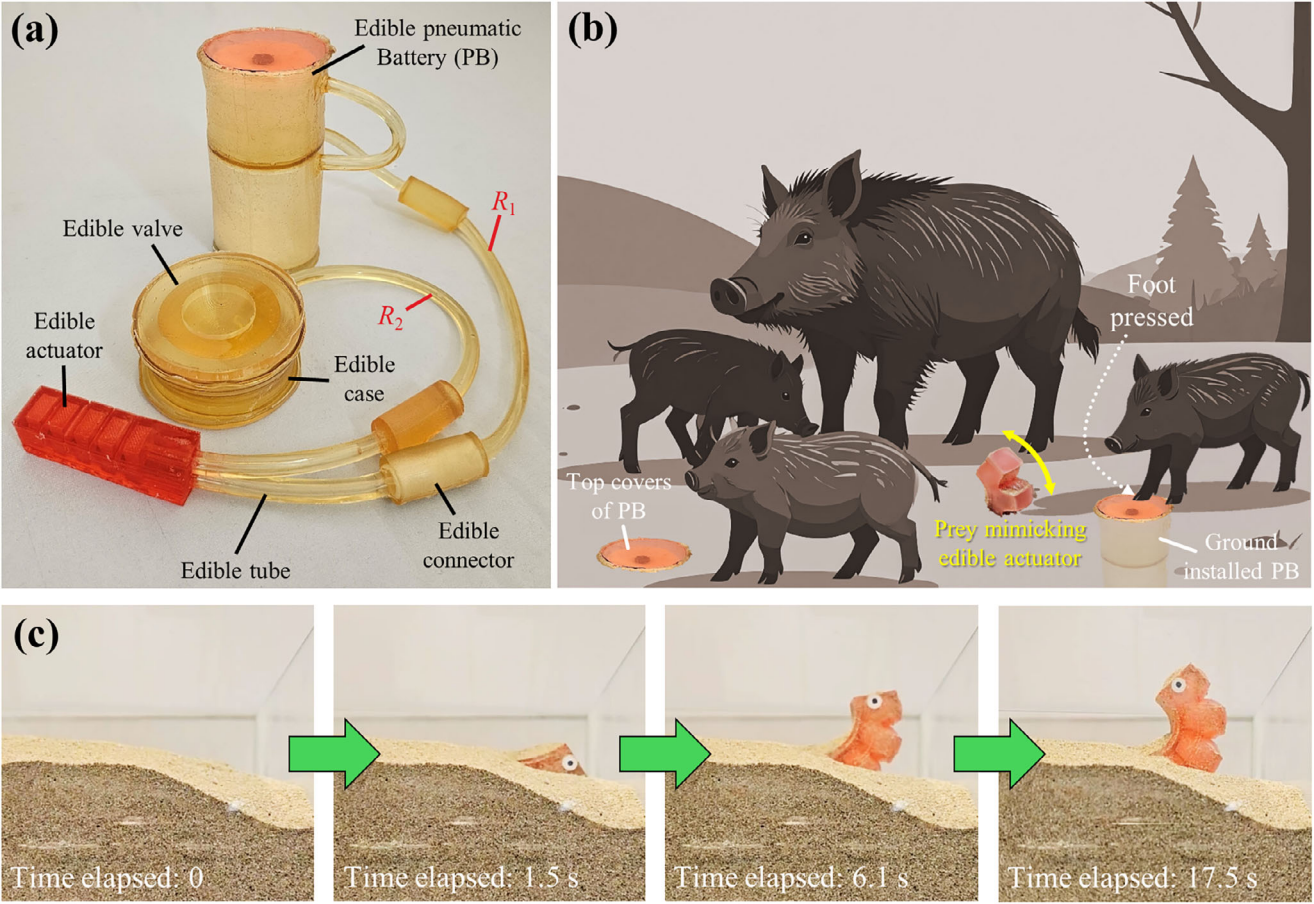
The implementation of fully edible system to power an edible pneumatic actuator when triggered. a) All the components are prototyped and assembled with edible materials, including fluidic resistance *R*_1_ and *R*_2_ that have an inner diameter of 0.7 mm (edible capsule size: large, CA concentration: 2.0 m, *D*_O_: 0.7 mm, *θ* = 100°, *t* = 9 mm). b) The proposed system has potential applications as a ground‐installable energy source that powers a prey‐mimicking edible actuator (infused with nutrients or medication), which is activated when wild animals step on the top cover of the pneumatic battery with sufficient foot pressure. In this context, wild boars are used as an illustrative example (created by Adobe Illustrator); however, the target species can be adjusted based on the specific application scenario. c) Bending motion of the edible actuator installed in sand (see also Video S5, Supporting Information).

We prototyped a fully edible system (**Figure** **5**a and see Experimental Section; Figure S17, Supporting Information, for more details) with parameters *D*_O_ = 0.7 mm, *R*_L1_ = 140 cm, and *R*_L2_ = 100 cm that showed the highest number of repeated actuations (Figure 4c). Since the inner diameter of the edible fluidic resistor was 0.7 mm, unlike the 1.0 mm used in the silicone tube (Figure 4), its length was reduced to 26% of the silicon tube to obtain equivalent fluidic resistance because the fluidic resistance of a circular channel is inversely proportional to the fourth power of its inner diameter.^[^ ^32^ ^]^ Specifically, the lengths of the edible fluidic resistances *R*_1_ and *R*_2_ are reduced to 36.4 cm and 26 cm, respectively.

Distributing consumable bait infused with life‐saving nutrients and vaccines is an effective strategy for controlling the spread of contagious diseases among wild animals.^[^ ^44^ ^]^ Studies have shown that carnivores and certain omnivores, such as wild boars, prefer to consume live, moving prey.^[^ ^42^, ^43^ ^]^ However, releasing live prey in open areas also increases the likelihood of attracting non‐target species (e.g., birds), which is not ideal for selectively attracting elusive animals like wild boars.^[^ ^48^ ^]^ We envision that our fully edible system could power animated robotic feed that can selectively attract such elusive species. For example, an animating bait infused with a swine fever vaccine could serve as a targeted delivery method for wild boars, which actively move in their home range for foraging.^[^ ^41^, ^45^ ^]^ This would involve fine‐tuning the size and threshold pressure of the pneumatic battery's top cover to match the characteristics of the target species, such as body weight and muscular force output. In this scenario, the edible actuator would mimic the movement of live prey when the pneumatic battery is activated by the foot pressure of heavy terrestrial animals, like wild boars (Figure 5b,c), generating a repeated bending motion to serve as an attractant (Video S5, Supporting Information). The number of repetitive motions is 8, with a range of motion of 17.95° ± 13.83°, and a motion period of 24.40 ± 17.73 s. Overall, the actuator motion in the fully edible system is more irregular compared to its counterpart shown in Figure 4. Unlike the screw‐lid method to fix the edible valve in Figure 4 (Figure S11, Supporting Information), gluing the edible valve to the edible case (Figure 5a) with edible gelatin requires at least 6 h to set (Figure S17, Supporting Information). During this time, the functionality of the edible valve may degrade (Figure S15, Supporting Information).

## 3 Discussion

This work presents an edible pneumatic battery and valve designed to power edible actuators with motion characteristics that can be programmed by adjusting fluidic resistance, chemical reactants and orifice size. The proposed system is also scalable to different sizes. The gas‐generating reaction is triggered by vertically pressing the center of the top cover, which in turn presses the push plug that breaks the blockage (refer to Figure 1b). However, applying this input load off‐center may not trigger the reaction (refer to Figure S18a,b, Supporting Information). The load required to break the blockage was measured as 26.14 ± 15.04 N (refer to Figure S18c, Supporting Information), where the variability was caused by imperfections of the capsule molding process with edible wax.

Our system has a lower gross mass (reagents plus packaging) than most compressors, canisters, or energy sources based on dry ice, which all require heavy metallic containers or batteries for operation (refer to **Table** **1**). While canister‐ and dry ice–based sources offer higher energy densities, our edible pneumatic battery performs comparatively to the chemical reaction–based systems. The energy density of our pneumatic battery is comparable to combustion‐based sources and is only lower than that of hydrogen peroxide, which is toxic. Flow rates are somewhat lower than those of compressors and canisters, making our system better suited for slowly powering pneumatic actuators. Finally, our system lasts longer than canister‐based sources and is comparable to other chemical systems, although it lasts less than biochemical ones. However, this operating time can be adjusted depending on the amount of reagents and pressurized gas. Overall, the proposed pneumatic battery achieves energy density and flow rates similar to those of most energy sources based on chemical reactions, with the added benefit of being fully biodegradable and edible.

**Table 1:** Comparison of existing pneumatic energy sources and the edible pneumatic battery.

Pneumatic energy source	Energy density[J g^−1^]	Flow rate [SLM]	Operation time	Fully edible	Gross mass [g]	Ref
Small CO_2_ canister + regulator (140 kPa)	N/A	N/A	45 s	No	194	[8]
Large CO_2_ canister + regulator (140 kPa)	N/A	N/A	240 s		666	
Small air compressor	72.6–99.3	1.7–3.5 SLM	N/A		130–171	[10]
Large air compressor	14.8–33.6	5.7–10.7 SLM	N/A		422–756	
Liquid CO_2_ canister (small size)	30.4–44.1	18–127 SLM	N/A		50–662	
Liquid CO_2_ canister (refillable)	58.1	300 SLM	N/A		1573	
Air canister (refillable)	27.1–83.6	350–650 SLM	N/A		909–1975	
Hydrogen peroxide	10–28.0	N/A	N/A		357	
Butane combustion	2.37	N/A	N/A		62.7	
Sodium bicarbonate + citric acid	4.6^a)^	0.4–1.1 SLM	>3 m		400^a^	[14, 23]
	2.2^b)^		>3 m		844.4^b^	
CO_2_ from urea hydrolysis reaction	0.21^c)^	N/A	>2 h		250^c^	[18]
Dry ice power cell	15.73	0.4 SLM^d)^	7.5 h		1030	[48]
	16.60	4 SLM^d)^	57 m			
	17.30	20 SLM^d)^	11 m			
	17.48	110 SLM^d)^	2 m			
Pneumatic battery; citric acid: 3.0 M	1.39–2.34	0.27–1.55 SLM	5–10 m	Yes	136	This work

N/A means that the corresponding data are not available in the literature. ‘Fully edible’ means that the packaging material is edible too. ‘Gross mass’ refers to the total mass of packaging material and included reagents. Please refer to Note S4 (Supporting Information) for the calculation of the energy density of the pneumatic battery. Figure notes:

^a)^ A bottle mass (before storing chemical reagents) is assumed to be 50 g.

^b)^ A bottle mass (before storing chemical reagents) is assumed to be 500 g.

^c)^ The total mass (the sum of chemical reagents and a 150 mL glass bottle) is assumed to be 250 g.

^d)^ Standard condition (20 °C, 1 atm) is assumed when converting liter per minute into SLM (standard liter per minute).

A soft robot should carry its own energy source to operate untethered. However, a high mass ratio of the energy source (*M*_E_ = mass of energy source/mass of entire robot) can hinder the robot's ability to perform locomotive tasks such as crawling or walking.^[^ ^8^ ^]^ In previously developed soft robots with on‐board energy sources, the *M*_E_ was 8.0% for a rolling robot (mass of pump and battery/mass of entire robot),^[^ ^7^ ^]^ and 14.5% for a legged robot (mass of compressed gas canister/mass of entire robot).^[^ ^8^ ^]^ In contrast, the *M*_E_ of the fully edible system shown in Figure 5 (mass of edible pneumatic battery/mass of entire system) is 51.5 %. While such a high *M*_E_ is acceptable for a stationary edible robot (e.g., Figure 5), it presents a challenge for powering mobile robots. Therefore, reducing the mass of the pneumatic battery is essential for enabling locomotion, which is discussed in the following paragraph.

When the orifice diameter (*D*_O_) is 1.5 mm, our edible pneumatic battery is able to dispense CA almost completely, leaving no significant residue (Figure S8, Supporting Information). However, when *D*_O_ is smaller than 1.5 mm, the mass ratio of residual CA (= the mass of CA remaining inside the edible capsule / the initial mass of CA before the reaction) can reach up to 50% (Figure S8, Supporting Information). Fixing D_O_ = 1.5 mm enables an edible pneumatic battery to carry a precise amount of CA and sodium bicarbonate (without excess chemicals), depending on the desired operation time. On the other hand, fixing *D_O_* = 1.5 mm requires the bottom compartment (Figure 1a) to be relatively tall, resulting in a bulkier pneumatic battery. This is because the reaction between CA and sodium bicarbonate produces foam (Figure S9, Supporting Information), which can obstruct CA release through the orifice if the bottom compartment is too low. The vertical height of the foam increases with both *D*_O_ and CA concentration (Figure S9, Supporting Information). Therefore, using a *D*_O_ smaller than 1.5 mm allows for a more compact and lightweight edible pneumatic battery, though this comes at the cost of leaving residual CA. In the end, the *M*_E_ of edible pneumatic battery can be reduced by 1) optimizing the capsule's design and material to ensure complete CA discharge even when *D*_O_ < 1.5 mm, and 2) developing an edible antifoaming agent to remove the necessity of keeping a higher bottom compartment when *D*_O_ is large.

Two noticeable modeling errors are observed in motion period (*T*_M_) when *D*_O_ = 0.9 mm, specifically at *R*_L1_ = 190 cm and *R*_L2_ = 50 cm, and at *R*_L1_ = 240 cm and *R*_L2_ = 0 (Figure 4e). However, the corresponding estimations of *ΔP*_a_ and *ΔP*_v_ remain accurate in both cases (Figure 4g,h). This indicated that the model tends to overestimate *T*_M_ by 20–30% when the gas production rate from the pneumatic battery is high (i.e., at large *D*_O_ values), and it may introduce additional errors when *D_O_* exceeds 0.9 mm. The slit valve is modeled as a bistable switch (see Figure 4f), which either blocks (slit closed) or releases gas (slit open) by toggling *R*_3_ between two finite resistance values (refer to Note S2, Supporting Information). This bistable representation provides a reasonable approximation of *T*_M_ when *D*_O_ is less than 0.9 mm. However, in reality, the slit valve does not operate in two discrete states. Its shape and the slit gap dynamically change during repeated gas release cycles.^[^ ^3^ ^]^ Therefore, the dynamic behavior of the edible valve under higher gas flow rate (i.e., when *D*_O_ ≥ 0.9 mm) needs to be further investigated to capture more rapid actuation scheme.

The top cover of the pneumatic battery must be physically pressed to initiate the chemical reaction. Other triggering methods could be used to enable self‐deployment of the system in outdoor scenarios. For example, replacing the top cover with a humidity‐responsive bistable actuator, which has a hygroscopic material on one side and an inert layer on the other side, could autonomously trigger the chemical reaction in a high‐humidity environment when the hygroscopic layer swells and is snap‐buckled toward the CA capsule.

Because the edible valve was manufactured with a gelatin hydrogel (see Experimental Section), it dehydrated and shrank over time. This caused the critical pressure of the gas release to change. Therefore, the stability of the valve material should be enhanced to ensure a longer lifespan and more predictable performance. This could be achieved by selecting alternative valve materials that prevent moisture loss and volumetric shrinkage, such as gelatin hydrogels infused with edible salts like choline chloride^[^ ^35^ ^]^ or edible ionic liquids, which are significantly less volatile than water.^[^ ^46^ ^]^ Alternatively, inert edible materials like beeswax could be applied by spray coating^[^ ^27^ ^]^ or directly mixed with gelatin hydrogels^[^ ^47^ ^]^ to increase hydrophobicity and minimize undesirable changes in material properties caused by moisture loss.

## 4 Experimental Section

### Material Usage

Gelatin (sourced from porcine skin, Sigma–Aldrich), glycerol (99%, Abcr), distilled water, beeswax (Sky Organics), carnauba wax (Armonia), citric acid (99.5%, Sigma–Aldrich), sodium bicarbonate (S5761, Sigma–Aldrich), and edible paper (Scrap Cooking) are used as received. All the silicone molds to manufacture an edible pneumatic battery are made of Smooth‐Sil 940 (Smooth‐On). Other molds to manufacture an edible valve and actuator are 3D‐printed with Tough PLA (Ultimaker). An inedible cylindrical case (see Figure S12, Supporting Information) to replicate the exterior case of the pneumatic battery in **Section** **2.4** was 3D printed with BioMed Clear 80A (Formlabs).

### Fabrication Method

Gelatin, glycerol, and distilled water are mixed in a 1:3:3 mass ratio in the fabrication of an edible pneumatic actuator. The same materials are mixed in 1:2:2 or 1:2.5:2.5 to fabricate an edible valve. An edible tube (including edible fluidic resistance) was manufactured with a 1:1:3 gelatin mixture. In the case of the cylindrical housing of the edible pneumatic battery (i.e., top and bottom compartments illustrated as a blue translucent cylinder in Figure 1a) and edible valve (see Figure S17, Supporting Information), gelatin and water were mixed in a 1:3 mass ratio without any glycerol to create stiff structures. Meanwhile, the top cover (of the pneumatic battery) to press the edible capsule was fabricated using a 1:0.35:3 gelatin mixture.

An edible capsule storing citric acid and an edible cylindrical case is molded to place the edible capsule and sodium bicarbonate inside. Please refer to Figure S1 (Supporting Information) to see a complete fabrication scheme. An edible valve and edible actuator are also molded, similar to the fabrication technique described in,^[^ ^29^, ^31^ ^]^ respectively. The slit was cut using a surgical blade with the aid of a slit cutting aligner (see Figure S17, Supporting Information), which helped maintain a consistent cutting orientation and size. An edible tube is molded by casting gelatin inside a plastic straw, while a 0.7 mm diameter nylon wire (for an edible fluidic resistance) or 2.5 mm diameter carbon rod (for general purpose connection) is aligned at the center. The connection between two edible tubes achieved by wrapping a water‐soaked edible paper around the joint, followed by reinforcement with gelatin. More details can be found in Figure S17 (Supporting Information).

### Experimental Method

The CO_2_ gas production rate and associated mass change during the citric acid and sodium bicarbonate chemical reaction were measured with a digital scale (KERN, 0.01 g resolution). Sodium bicarbonate powder is placed inside an inedible cylindrical case, while an empty edible capsule is placed above the powder (see Figure S1, Supporting Information). Note that all of these are placed on the scale. A liquid citric acid is poured inside the edible capsule to initiate the chemical reaction. The mass data was collected every second via serial communication between the scale and a computer via software (Balance Connection) provided by the scale manufacturer. All the mass data are processed with Matlab (MathWorks) without any filtering and used to produce plots in Figure 2. The foam height during the chemical reaction was measured by video filming the reaction with a mobile phone (Galaxy Z Flip5), while a ruler was placed next to the reaction cylinder as a scale bar.

The critical pressure (*P*_C_) of the edible valve was measured with a pressure sensor (MPX5700AP), while pressurized air was manually supplied by a syringe (volume: 120 mL, inner diameter: 38.5 mm). The edible valve was tightly fixed on a cylindrical case that had an internal volume of 140 mL. The schematic of the *P_C_* measurement setup is illustrated in Figure S11 (Supporting Information).

The edible actuator's self‐repetitive motion data was recorded with the same smartphone in 30 frames per second. The two markers attached to the actuator were traced with video analysis software (Tracker), and the bending angle was obtained. Simultaneously, the actuator and valve's pressure changes were measured with two pressure sensors (MPX5700AP) at a 30 Hz data acquisition rate with an Arduino Uno microcontroller.

The force required to break the blockage was measured by manually pressing the top cover with a pillar coupled to a force sensor (ATI Nano 17), as illustrated in Figure S18c (Supporting Information). An Edible capsule was placed underneath the top cover, and the measurement was repeated six times.

Statistical Analysis: All experiments were performed independently, and the number of samples (*n*) is specified in the figure captions. No pre‐processing was done to the experiment data unless otherwise specified. The statistical significances of *D*_O_ and *R*_L1_ (or *R*_L2_; note that *R*_L1_ + *R*_L2_ = 240 cm) were analyzed by two‐way ANOVA (Analysis of variance) in Figure 4d,e. Results were presented as mean ± standard deviations by using MATLAB R2025a software.

## Conflict of Interest

The authors declare no conflict of interest.

## Supporting information



Supporting Information

Supplemental Video1

Supplemental Video2

Supplemental Video3

Supplemental Video4

Supplemental Video5

## Data Availability

The data that support the findings of this study are available in the supplementary material of this article.
