# Supplementary material for: Edible Pneumatic Battery for Sustained and Repeated Robot Actuation
Source: Adv Sci (Weinh). 2025 Oct 24;13(15):e09350. doi: 10.1002/advs.202509350 (PMC13042464; doi:10.1002/advs.202509350)
Supplement: Supplementary file 1 — Supporting Information [file ADVS-13-e09350-s003.docx]

**Supporting Information**

Edible Pneumatic Battery for Sustained and Repeated Robot Actuation

*Bokeon Kwak^1^, Shuhang Zhang^1^, Alex Keller^2^, Qiukai Qi^2^, Jonathan Rossiter^2^, Dario Floreano^1*^ (*corresponding author)*

^1^ B. Kwak, S. Zhang, D. Floreano

Address: Laboratory of Intelligent Systems, School of Engineering, Ecole Polytechnique Federale de Lausanne (EPFL), 1015 Lausanne, Switzerland

* Email: [dario.floreano@epfl.ch](mailto:dario.floreano@epfl.ch)

^2^ A. Keller, Q. Qi, J. Rossiter

Address: School of Engineering Mathematics and Technology, University of Bristol, Bristol, UK.


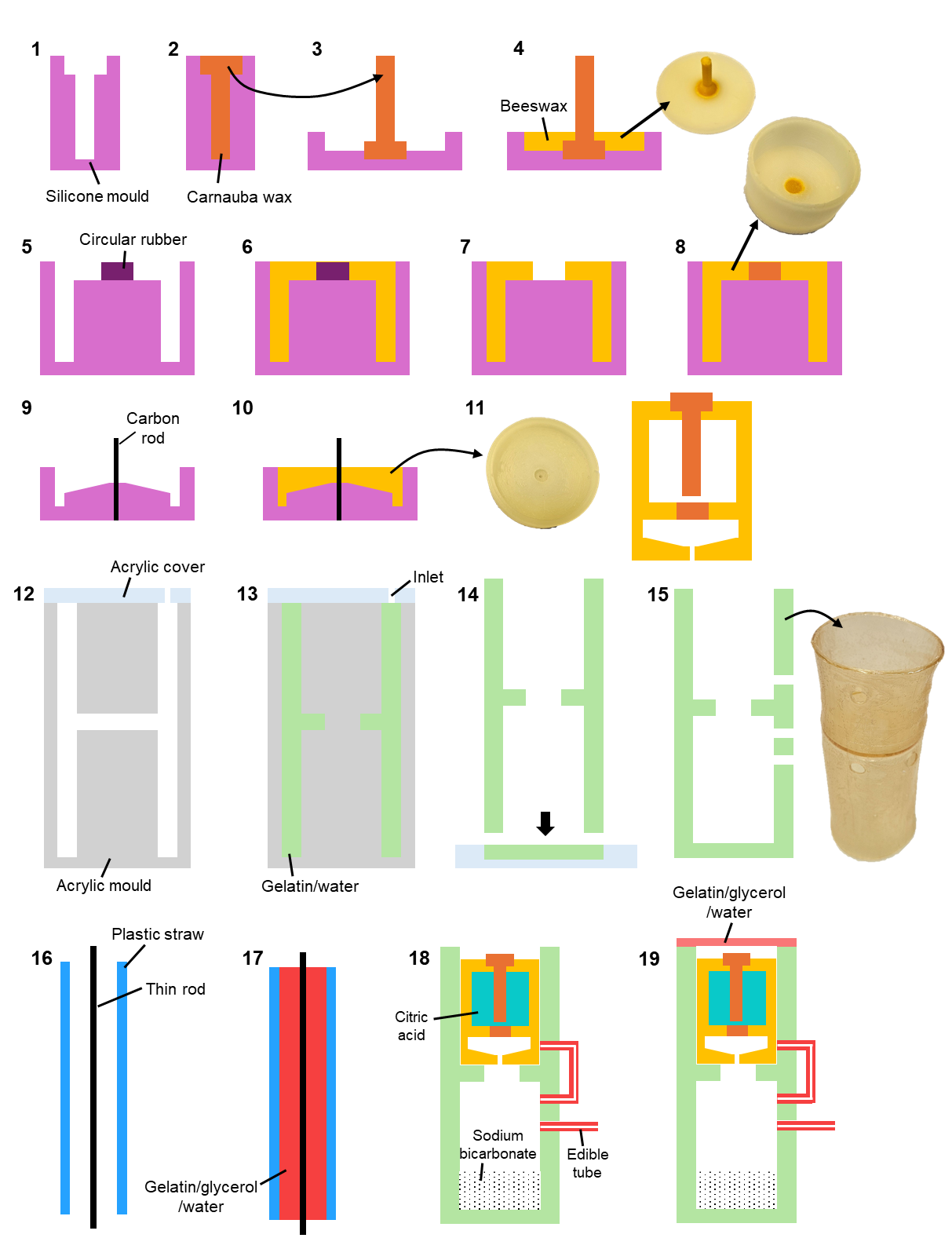


**Figure S1**. Fabrication process of edible pneumatic battery. Gelatin material ratios are summarised in Table S3. **(1)**: preparation of silicone mould (material: Smooth-Sil^TM^ 940) of the push plug, **(2)**: pour melted carnauba wax and let it cool down at room temperature, **(3)**: demould the push plug and vertically align it on a silicone mould, **(4)**: pour melted beeswax and let it solidify at room temperature, **(5)**: preparing a silicone mould of the edible capsule, **(6)**: pour melted beeswax, **(7)**: remove the silicone ring after solidification, **(8)**: pour melted carnauba wax at the center, **(9)**: preparing a silicone mould of the orifice surface and aligning a carbon rod (to shape an orifice of diameter *D_O_*) at the center, **(10)**: pour a melted beeswax, **(11)**: demoulding and gluing with the parts obtained from step (4) and (8) by using melted beeswax as a glue, **(12)**: prepare an acrylic mould of the pneumatic battery container, **(13)**: pour gelatin/water mixture (1:3 mass ratio) through the inlet, **(14)**: casting another gelatin/water layer and gluing it with the container, **(15)**: punch three holes, **(16)**: align a thin rod at the center of the plastic straw, **(17)**: pour gelatin/glycerol/water mixture (1:1:3 mass ratio) inside the straw, **(18)**: connect the edible tubes from step (17) with the battery container, then put sodium bicarbonate and the edible capsule pre-injected with citric acid, **(19)**: gluing a circular gelatin/glycerol/water (1:0.35:3 mass ratio) layer (thickness: 2 mm) on top of the container by using the same gelatin/glycerol/water mixture as a glue.


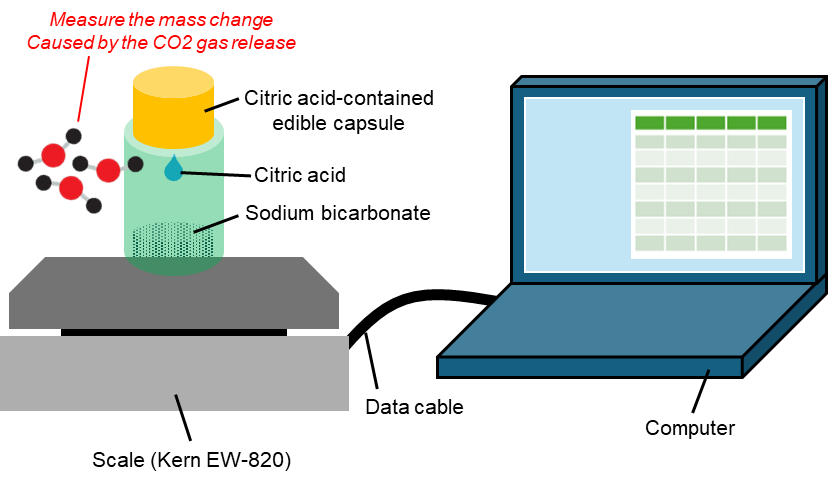


**Figure S2**. The experiment is set up to measure the mass change (i.e., CO_2_ gas loss) of the chemical reaction between citric acid and sodium bicarbonate. Total mass is measured every 1 second, and mass data is transferred to the computer for analysis.


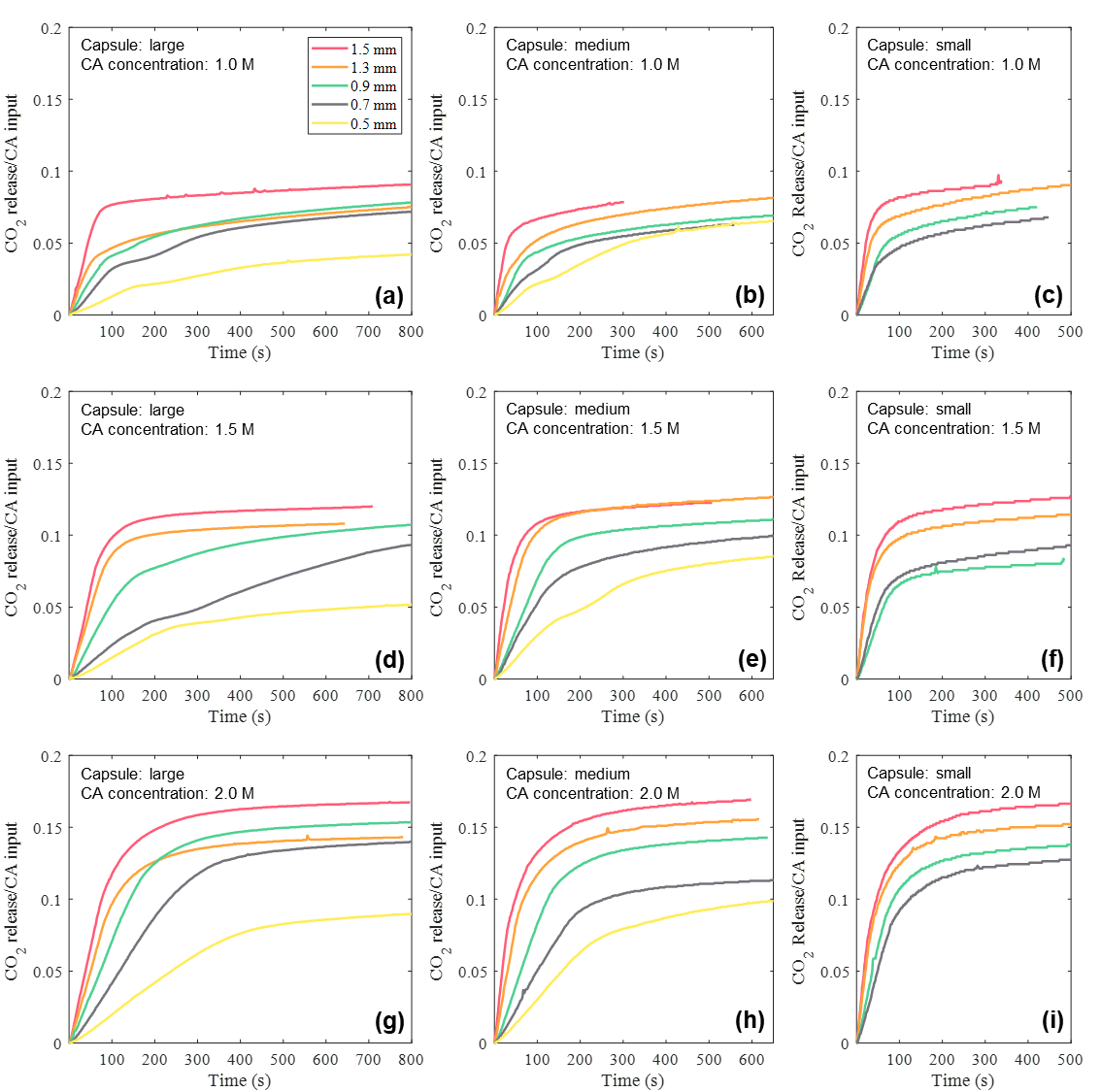


**Figure S3**. CO_2_ gas release during the chemical reaction normalized by the corresponding citric acid (CA) input mass. Note that all the graphs share the same color legend, which describes the diameter of the orifice *D_O_*, shown in (a). **(a – c)**: large, medium, and small capsule sizes, respectively, when CA concentration is 1.0 M. **(d – f)**: large, medium, and small capsule sizes, respectively, when CA concentration is 1.5 M. **(g – i)**: large, medium, and small capsule sizes, respectively, when CA concentration is 2.0 M.


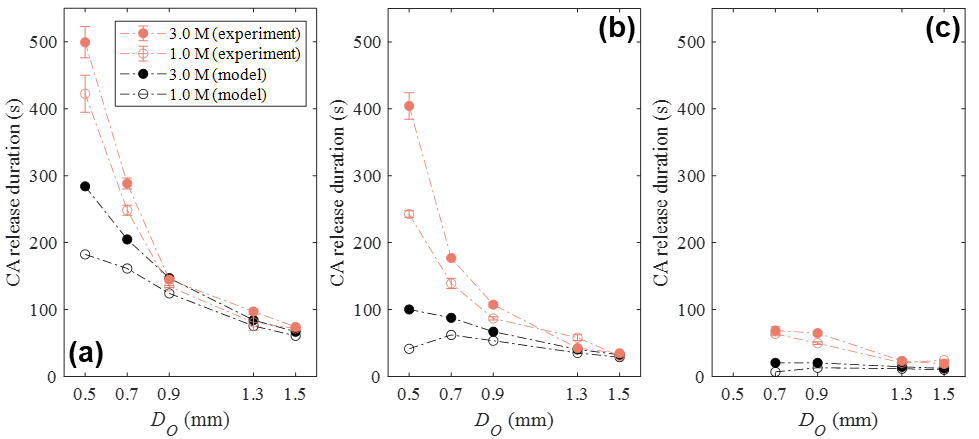

**Figure S4**. The required time to complete the citric acid release from edible capsules (**a**: large, **b**: medium, **c**: small). The citric acid release duration for 1.5 M and 2.0 M concentrations falls between 3.0 M and 1.0 M (data are not shown). The corresponding model estimations (see Supplementary Note 1) are depicted with black filled markers (3.0 M) and black empty markers (1.0 M). The marker legend shown in (a) is applied to both (b) and (c) in the same manner. Each case was tested three times (n = 3).


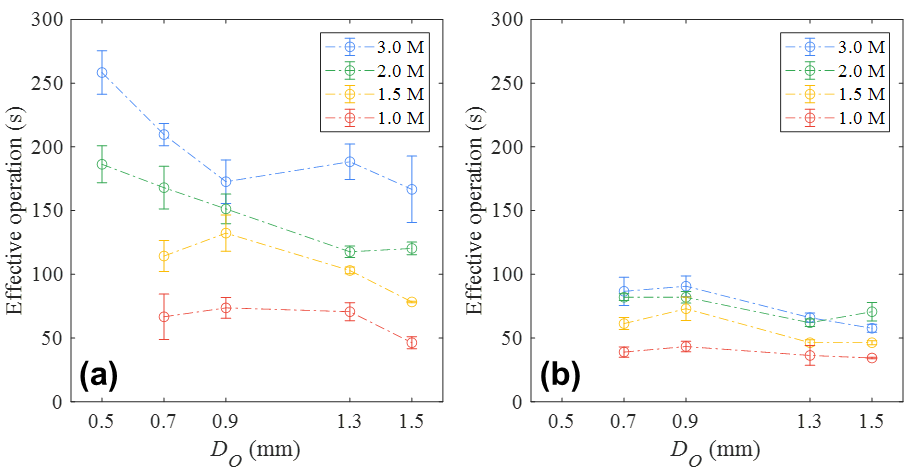

**Figure S5**. The effective operation time (i.e., the elapsed time until the CO₂ release rate drops below 10% of the maximum flow rate of a commercial microcompressor [10]) of the pneumatic battery as a function of citric acid concentrations (1.0 – 3.0 M) and orifice diameter *D_O_* (**a**: medium capsule, **b**: small capsule). Please refer to Fig. 2d for the effective operation measured from the large capsule. Each case was tested three times (n = 3).


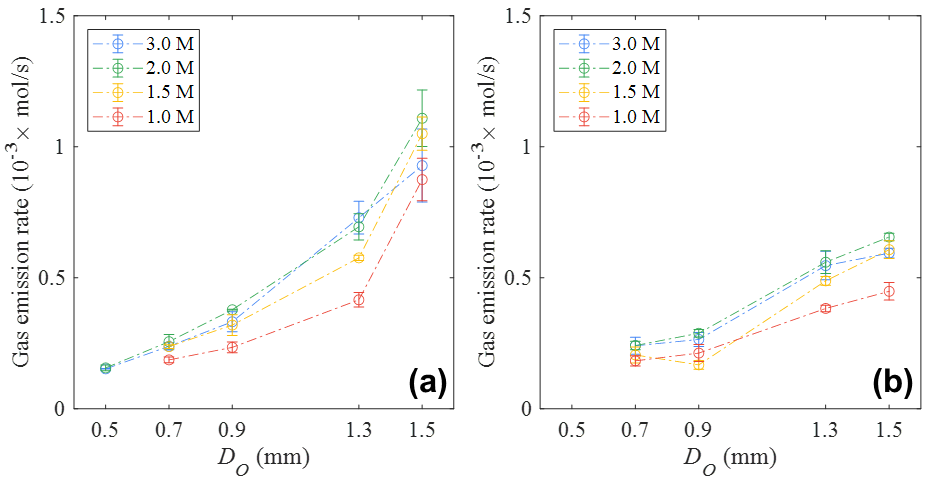

**Figure S6**. The CO_2_ release rate during the initial phase of the reaction, when the CO_2_ release vs time graphs (see Fig. S3 and Fig. 2a-c) are exhibiting a linear increase (**a**: medium capsule, **b**: small capsule). Please refer to Fig. 2a-c for the gas emission rate obtained from the large capsule. Each case was tested three times (n = 3).


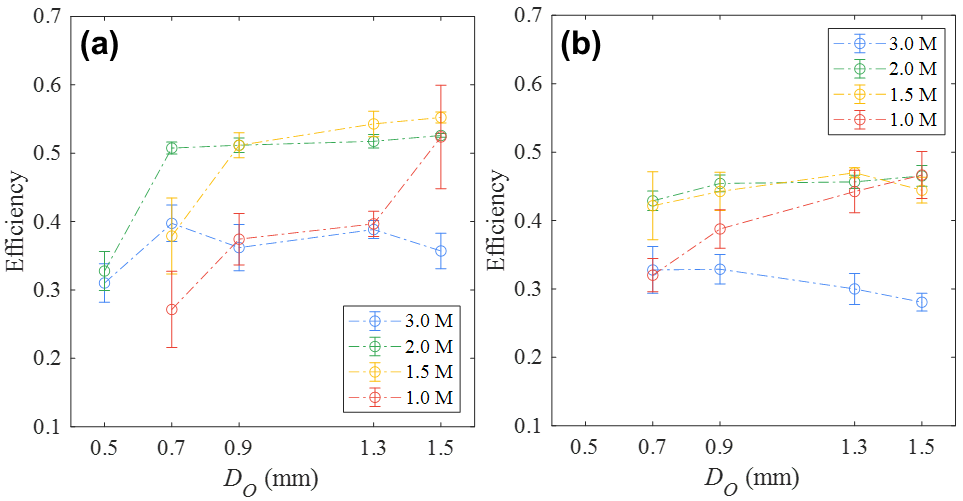

**Figure S7**. Comparison of efficiency: the total amount of CO₂ released during effective operation, divided by the maximum amount of CO₂ released if all the citric acid (discharged from the capsule) reacted with sodium bicarbonate (**a**: medium, **b**: small). Please refer to Fig. 2f for the efficiency of a large capsule. Each case was tested three times (n = 3).


**Figure S8**. Citric acid remained inside the capsule (normalized with the initial mass of citric acid stored inside the capsule) after the gravity-driven discharge is complete. Since the concentration-wise differences of the residual citric acid are minor, they were combined as long as their orifice diameter (*D_O_*) and capsule size are the same. Each case was tested three times (n = 3).


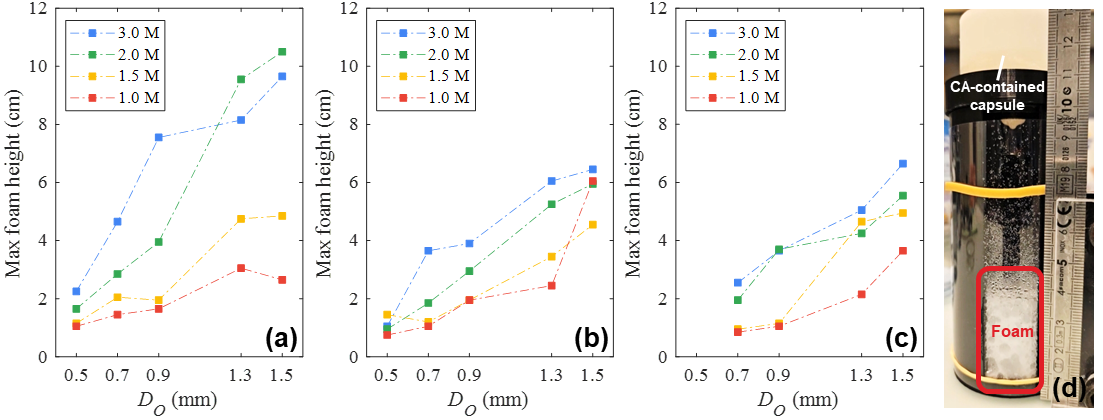

**Figure S9**. The maximum height of the foam generated during the chemical reaction of citric acid (CA) and sodium bicarbonate (**a**: large capsule, **b**: medium capsule, **c**: small capsule). **(d)**: snapshot of the foam generated when mixing 1.5 M concentration of citric acid (dispensed from a medium-sized capsule with orifice diameter *D_O_* = 1.5 mm) and sodium bicarbonate. Each case was tested five times (n = 5).


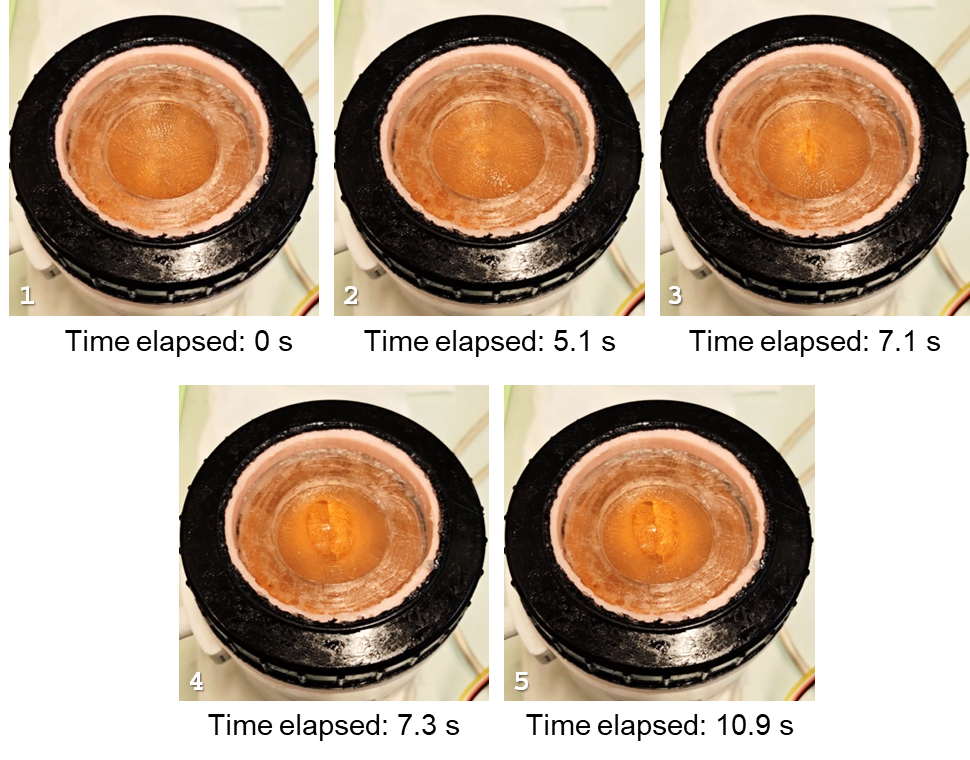

**Figure S10**. The sequence of slit opening located at the center during the manual pressurization with a syringe. Refer to Fig. S11 for more details of the test setup. **(1)**: before pressurization, **(2)**: after 5.1 s the valve is start being pressurized and bent upward, while the slit is closed, **(3)**: the slit is about to be opened upon continuous pressure input, **(4)**: the slit is opened and pressurized air inside is released, **(5)**: the slit remains opened in the presence of pressure input.

**
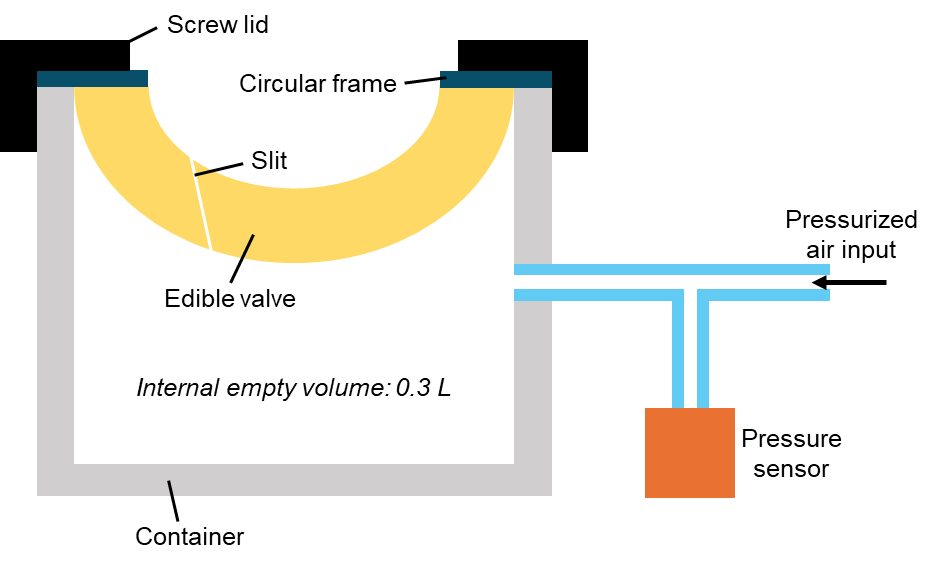
**
**Figure S11**. The experiment is set up to measure the critical pressure (*P_C_*) of an edible valve upon continuous pressurized air input from a syringe. Pressure was measured with a pressure sensor (MPX5700AP) at a 30 Hz rate. Please refer to Materials and Methods for more details.

**
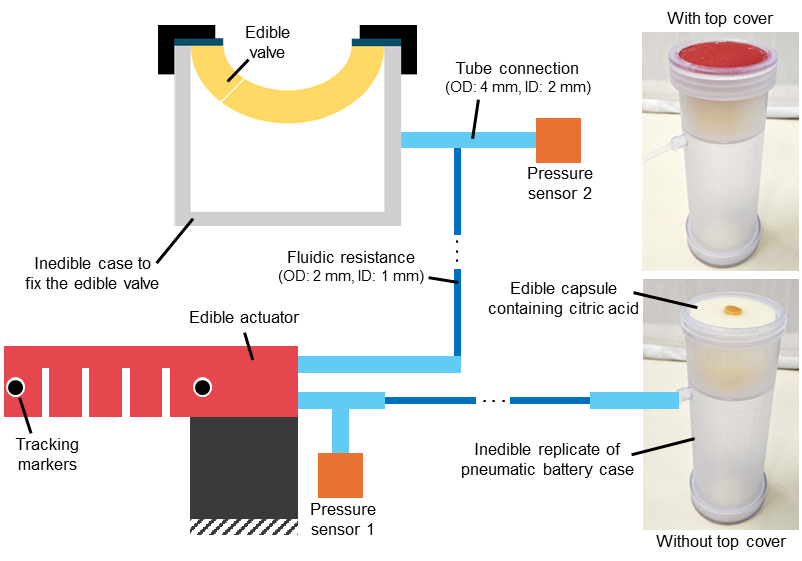
**
**Figure S12**. The experiment setup is used to characterize the self-repetitive motion of the edible actuator driven by a partially edible pneumatic battery (the case housing the edible capsule and sodium bicarbonate, and the top cover are made with inedible materials for efficient repetition of the experiment) and edible valve (the case holding the edible valve remains inedible to replace the edible valve easily). All the tube connections and fluidic resistances are done with silicone tubes, where OD and ID refer to their outer diameter and inner diameter, respectively. Pressure sensors 1 and 2 measured the pressure change of the edible actuator and the valve, respectively.


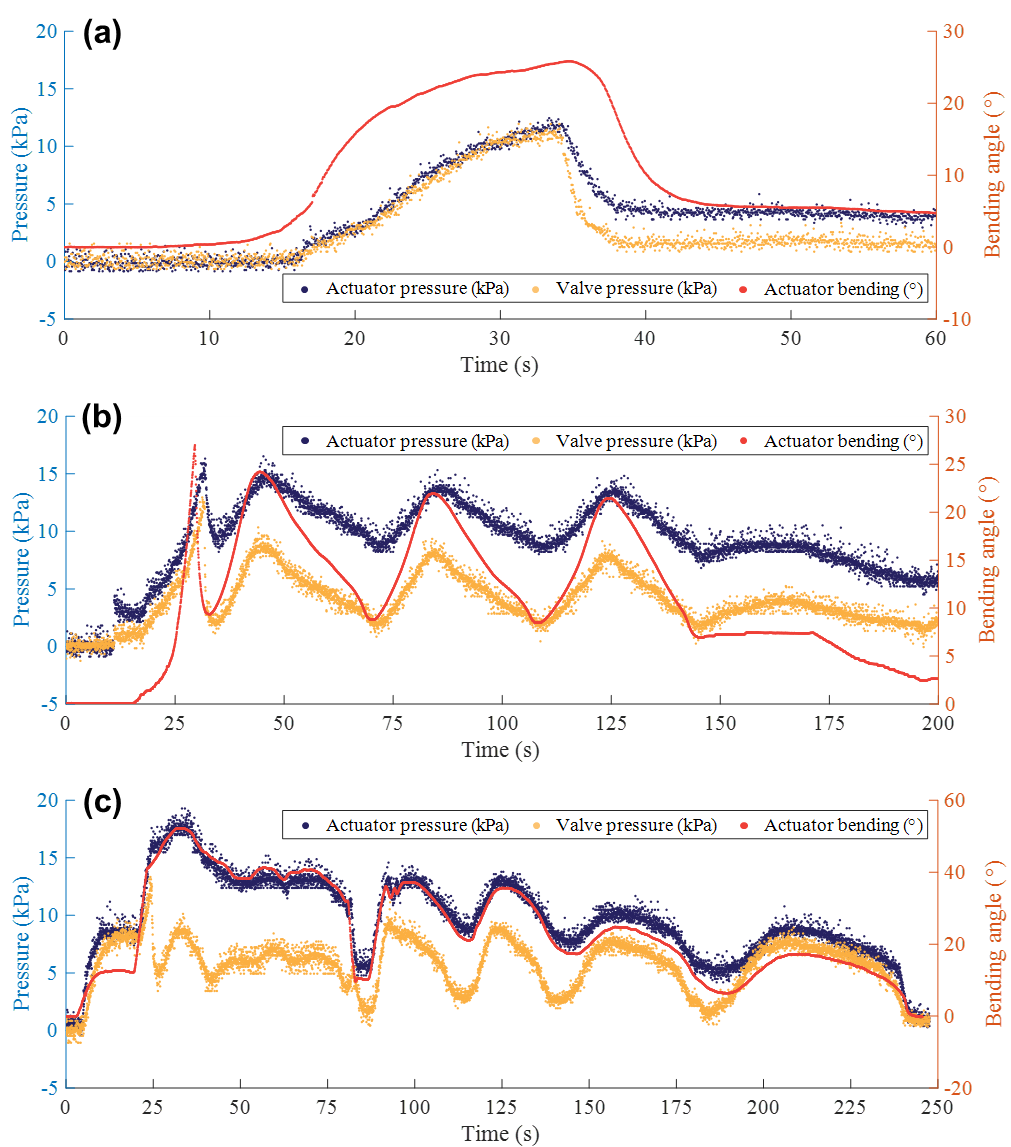

**Figure S13**. The change of pressure and the bending angle of the actuator when small and medium-sized capsules were used to discharge citric acid. **(a)**: Small capsule with *D_O_* = 0.9 mm, edible valve; *t* = 8 mm, *θ* = 100°, fluidic resistance; *R_L_*_1_ = 60 cm, and *R_L_*_2_ = 80 cm. The maximum number of repetitive motions of the edible actuator was one, even with other combinations of fluidic resistance. **(b)**: Medium capsule with *D_O_* = 0.7 mm, edible valve; *t* = 8 mm, *θ* = 100°, fluidic resistance; *R_L_*_1_ = 250 cm, and *R_L_*_2_ = 90 cm. The number of repetitive actuator’s motion is four. **(c)**: Medium capsule with *D_O_* = 0.9 mm, edible valve; *t* = 9 mm, *θ* = 100°, fluidic resistance; *R_L_*_1_ = 60 cm, and *R_L_*_2_ = 180 cm. The repetitive motion of the actuator was irregular, and its repetition was counted as seven.

**
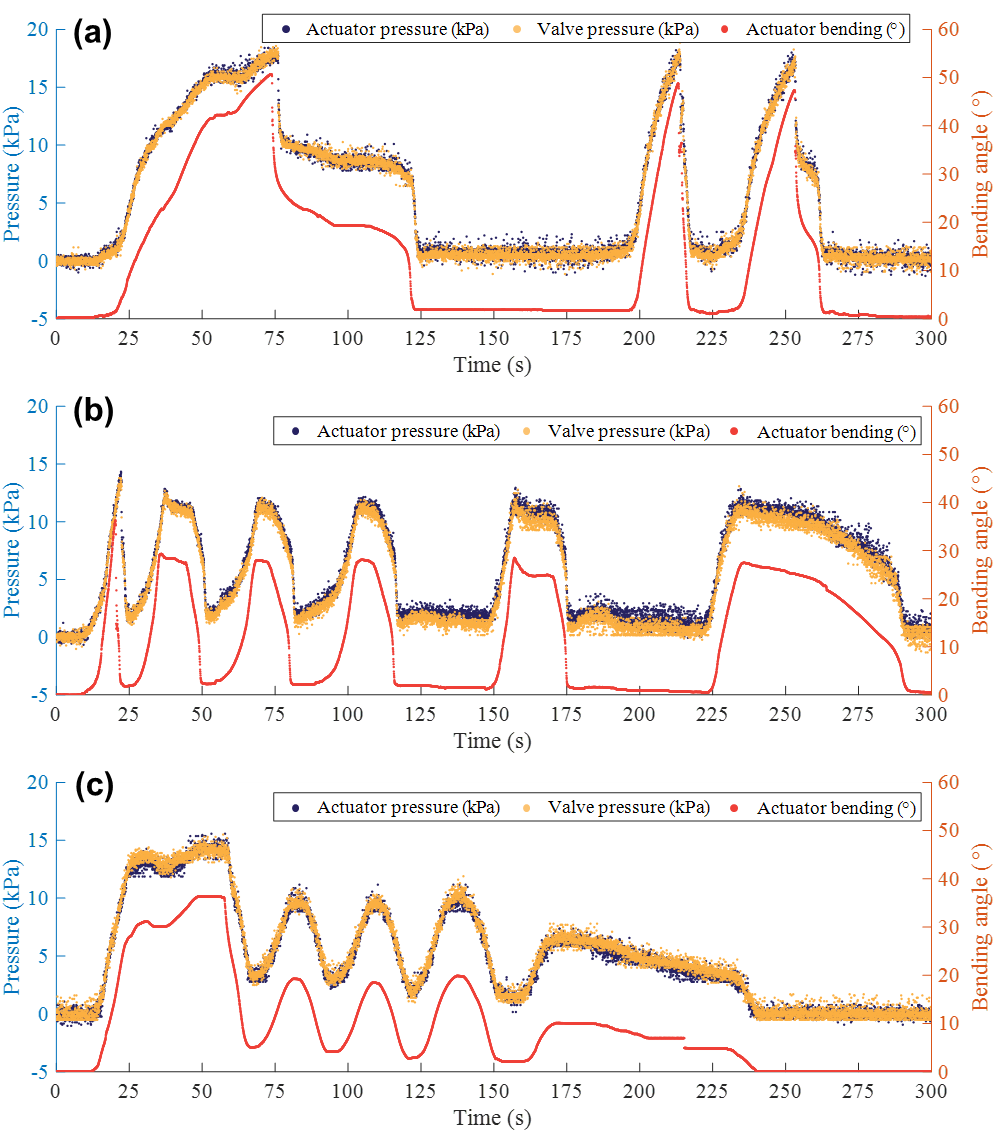
**
**Figure S14**. The change of pressure and the bending angle of the actuator when downstream fluidic resistance (*R*_2_ in Fig. 1c) is zero (**a**: *D_O_* = 0.5 mm, **b**: *D_O_* = 0.7 mm, **c**: *D_O_* = 0.9 mm). The size of the pneumatic battery capsule was large, and the edible valve type was characterized as *t* = 9 mm, *θ* = 100°.


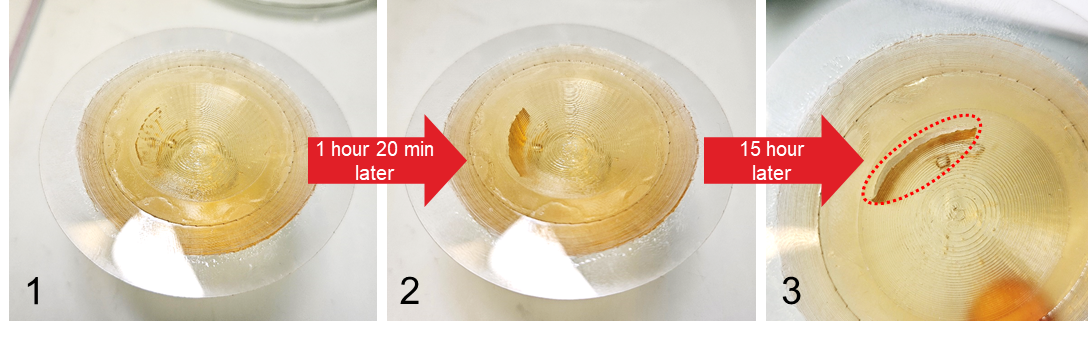

**Figure S15**. The gap of the slit valve is increased during the storage in room environment (temperature: 22°C, humidity: 35%). **(1)**: right after cutting the slit with a surgical blade, **(2)**: the gap of the slit starts getting increased, which is distinguished by the brownish area, caused by light refraction, **(3)**: the gap is noticeably increased and the slit valve cannot hold any pressure.


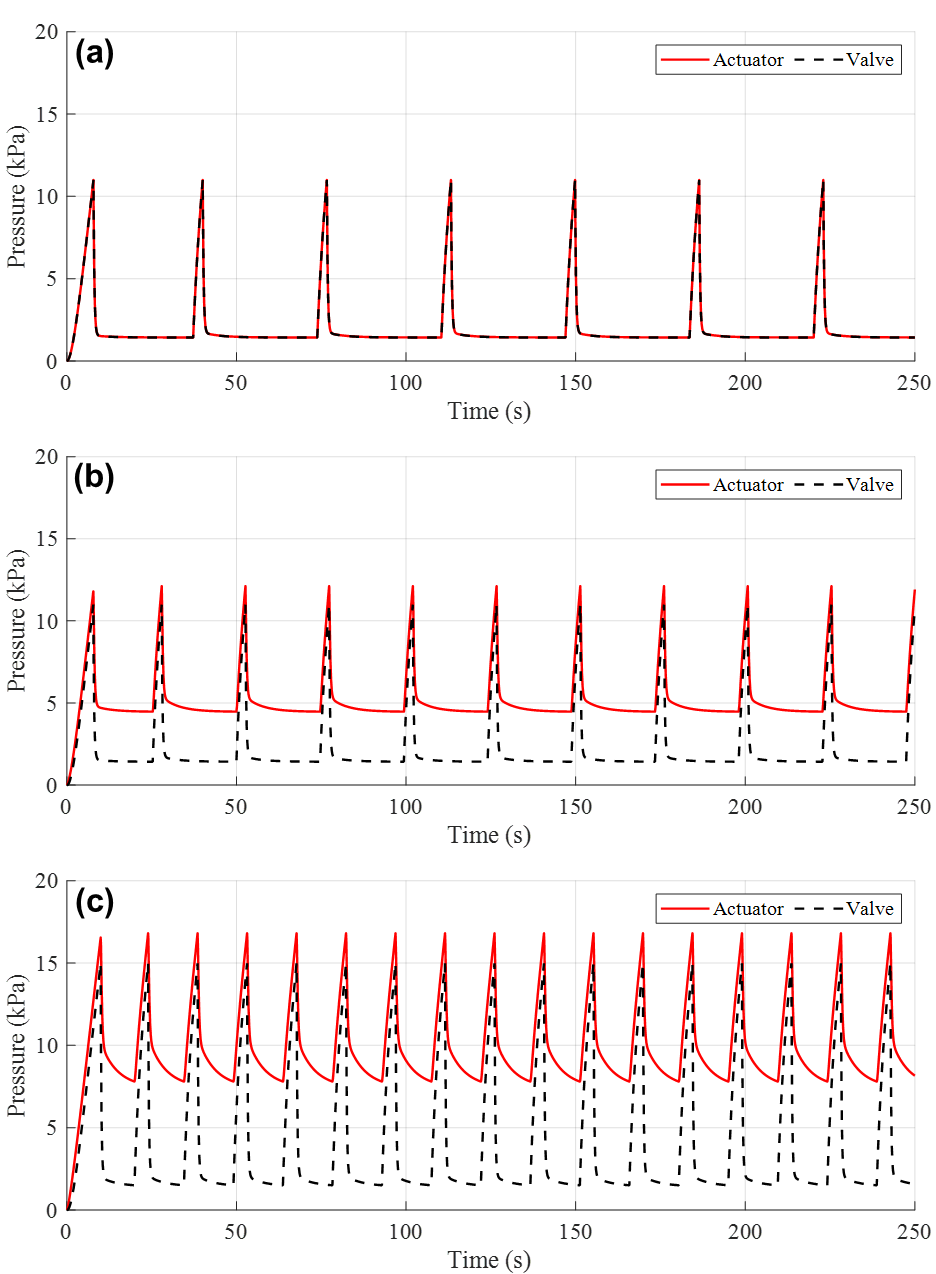

**Figure S16**. The simulated pressure change of the edible actuator and valve (**a**: *R_L_*_1_ = 240 cm, *R_L_*_2_ = 0, **b**: *R_L_*_1_ = 190 cm, *R_L_*_2_ = 50 cm, **c**: *R_L_*_1_ = 140 cm, *R_L_*_2_ = 100 cm). The number of pressure changes occurring within a time window is the number of repetitive actuations. More details of the simulated model can be found in Supplementary Note 2. The size of the pneumatic battery capsule was large (orifice size *D_O_* = 0.7 mm), and the edible valve type was characterized as *t* = 9 mm, *θ* = 100°.
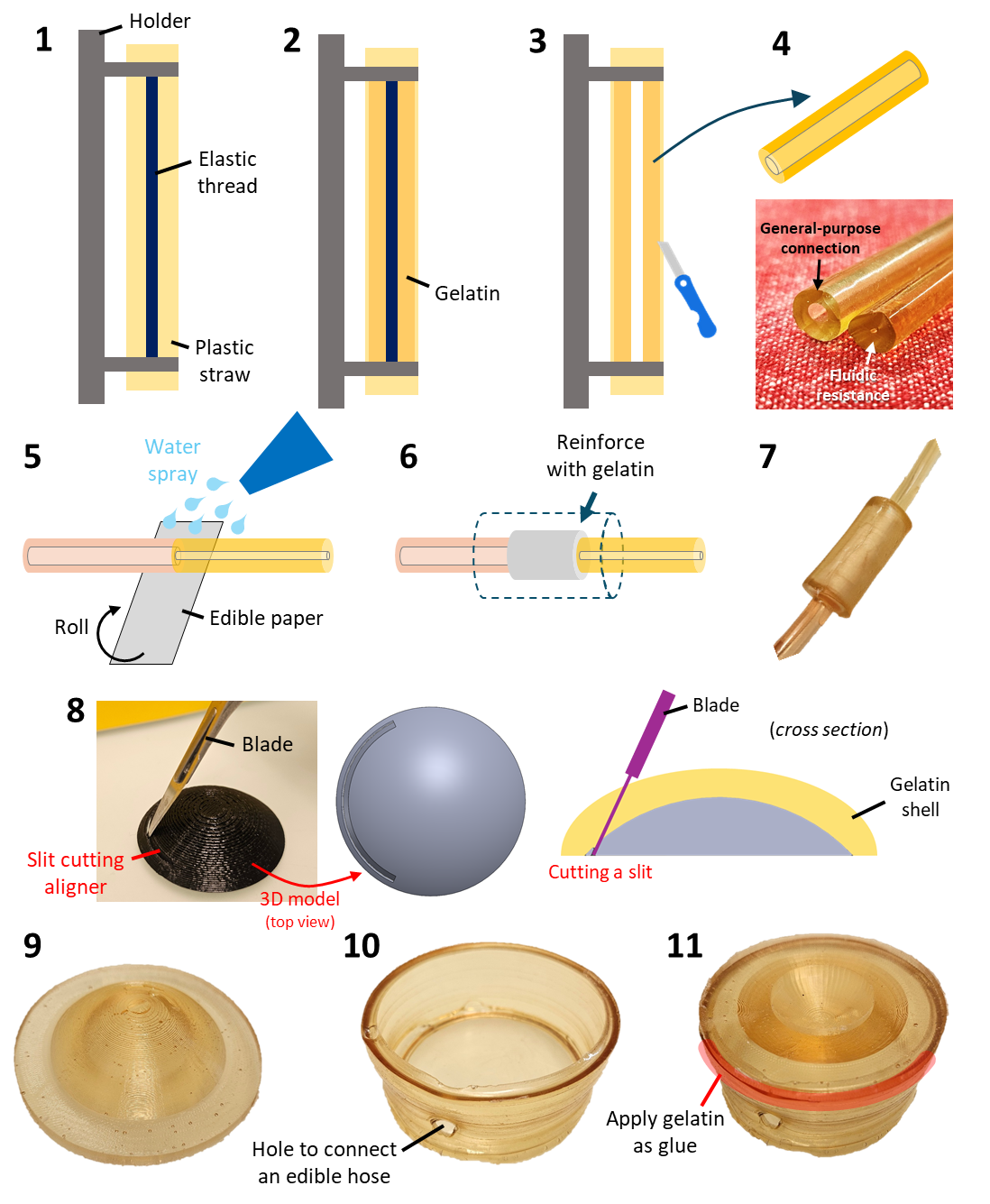

**Figure S17**. The fabrication process of the edible fluidic resistance and assembly process for a fully edible system shown in Fig. 5a. **(1)**: align an elastic thread (diameter: 0.7 mm) at the center of a plastic straw, **(2)**: cast a gelatin mixture (gelatin:glycerol:water = 1:1:3 mass ratio) and let it solidify, **(3)**: remove the straw and the elastic thread, **(4)**: let it dry in a room humidity environment at least for one day (general purpose connection tube: inner diameter of 2 mm, fluidic resistance: inner diameter of 0.7 mm), **(5)**: connection between the tube outlet from an actuator or pneumatic battery with the fluidic resistance was done by wrapping an edible paper while spraying some water to utilize the stickiness from the wet edible paper, **(6)**: the connection area wrapped with the edible paper was reinforced with extra gelatin (gelatin:water mass ratio; 1:3) to ensure air-tight sealing, **(7)**: complete connection between two edible tubes, **(8)**: place a gelatin shell (refer to Fig. 3a) on a slit cutting aligner and cut a round slit using a surgical blade by moving the blade along the aligner, **(9)**: an edible valve was fabricated by the same moulding method in [29], **(10)**: an edible case to hold the edible valve was moulded with the same method illustrated in Fig. S1, **(11)**: gelatin hydrogel in liquid state (heated at 70°C) is applied as an edible glue to fix the edible valve on top of the case.


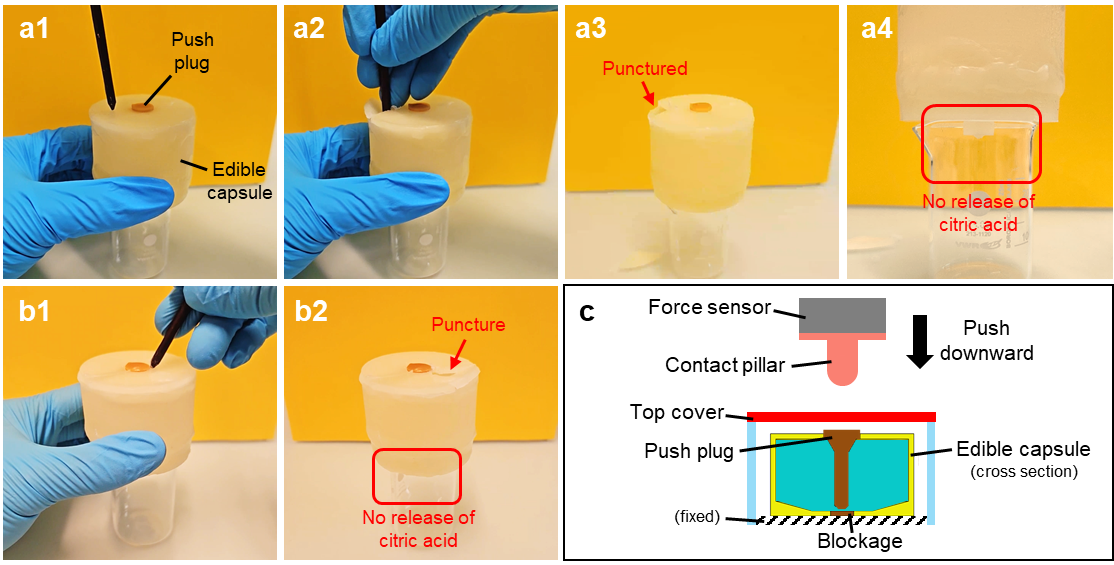


**Figure S18**. **(a1 – a2)**: Input load is applied off the center of the edible capsule filled with citric acid, **(a3)**: the side of the edible capsule is punctured, **(a4)** no release of the citric acid, as the blockage (see figure panel c or Fig. 1a in the manuscript) is still intact. **(b1)**: Input load is applied at an oblique angle close to the center, **(b2)**: the top surface next to the push plug is punctured, but the citric acid is not released. **(c)**: The experiment setup to measure the required force to break the blockage by vertically pressing the top cover (corresponds to ‘top cover’ in Fig. 1a of the manuscript) at the center. The measured force to break the blockage was 26.14 ± 15.04 N (*n* = 7).

**Supplementary Note 1**

Release of citric acid from an edible capsule can be initiated when the hydrostatic pressure (*P_s_*) at the orifice (diameter: *D_O_*; refer to Fig. 1a) is greater than the capillary pressure (*P_γ_*) around the orifice.

$$P_{\gamma}=\frac{2\gamma\cos\theta}{r}, P_{s}=\rho gH$$

where *γ* is surface tension of citric acid, *θ* is the contact angle between the citric acid and the edible capsule orifice (beeswax), *r* (= 0.5*D_O_*) is the radius of the orifice, *ρ* is the density of citric acid, *g* is gravitational acceleration constant (9.81 m/s^2^), and *H* is the height difference of citric acid contained in the edible capsule ^[S1]^; here *H* is 25.7 mm, 20.5 mm, and 14 mm, respectively for a large, medium, and small edible capsule.

This work assumes *θ* is 110° based on the experiment data ^[27]^. Meanwhile, *γ* and *ρ* of citric acid of 3.0 M, 2.0 M, 1.5 M, and 1.0 M concentrations are found from literature; *γ* = 65.49, 66.62, 67.27, 68.43 mN/m (from 3.0 M to 1.0 M), *ρ* = 1287.67, 1184.51, 1135.02, 1083.54 kg/m^3^ (from 3.0 M to 1.0 M) ^[S2]^. Thus, *P_γ_* and *P_s_* are obtained in Table S1 and S2. It is assumed that citric acid is not discharged through the orifice when *P_γ_* is greater than the corresponding *P_s_*. This explains why a small capsule with a 0.5 mm diameter orifice could not discharge citric acid in the experiment. The rest of the cases can spontaneously discharge citric acid, once the blockage (see Fig. 1a) is broken, but the discharge is assumed to be seized when *P_γ_* = *P_s_*, as *H* is decreased to critical height *h_c_*. In other words, *P_γ_* and *P_s_* are in equilibrium when *H* is reduced to *h_c_* after a certain amount of citric acid discharge.

The required time (*τ*) to discharge citric acid from *H* to *h_c_* is calculated as ^[25]^:

$$\tau=\frac{1}{C_{d}}\frac{A}{A_{O}}\sqrt{\frac{2}{g}}\left( \sqrt{H}-\sqrt{h_{c}} \right)$$

where *A* is the internal cross-section area of the capsule (i.e., π(*D_c_* - 2*t*)^2^/4; *t* is wall thickness as shown in Fig. 1a), *A_O_* is the surface area of the orifice (i.e., *πr*^2^), and *C_d_* is a discharge coefficient defined as ^[26]^:

$$C_{d}=0.611\left[ 87\left( \frac{\mu}{D_{O}\sqrt{gH}} \right)^{1.43}+\left( 1+\frac{4.5\mu}{D_{O}\sqrt{gH}} \right)^{-1.26} \right]^{-0.7}$$

where *μ* is the kinematic viscosity of citric acid and is obtained by dividing dynamic viscosity data from a literature ^[28]^ with corresponding density. As a result, the theoretical estimates of citric acid release time in Fig. S4 (black markers) are obtained from the equation *τ* above.

**Supplementary Note 2**

The system of equations to simulate the model in Fig. 4f is based on ideal gas law ^[22]^:

$$PV=nRT$$

where *P* is pressure, *V* is gas volume, *n* is number of moles of gas, *R* is the universal gas constant (i.e., 8.3145 m^3^⋅Pa⋅K^-1^⋅mol^-1^), and *T* is temperature (i.e., 293 K; room temperature). By letting *P*_atm_ = 101 kPa as an atmospheric pressure, the pressure at nodes 1, 2, and 3 (refer to Fig. 4f) is found from the following equations:

$$P_{1}=\frac{n_{1}RT}{V_{1}}-P_{\mathrm{atm}}, P_{2}=\frac{n_{2}RT}{V_{2}}-P_{\mathrm{atm}}, P_{3}=\frac{n_{3}RT}{V_{3}}-P_{\mathrm{atm}}$$

where *n*_1_, *n*_2_, and *n*_3_ refer the CO_2_ gas molecules (in moles) in an edible pneumatic battery, an edible actuator, and a case housing an edible valve, respectively, and *V*_1_, *V*_2_, and *V*_3_ are internal volume of an edible pneumatic battery, internal volume of an edible actuator, and internal volume of an edible valve housing the valve, respectively. Here, *V*_2_ is assumed to be linearly changed upon pressure input:

$$V_{2}=V_{2,i}+\Delta_{VP}P_{2}$$

where *V*_2,_*_i_* is the internal volume of the edible actuator without pressure input, and Δ*_VP_* is the volumetric increase rate of the actuator’s internal volume upon pressure input, which is considered as 3.2353×10^-10^ m^3^/Pa based on experiment data. We assume there is a virtual fluidic resistance through a slit valve (*R*_3_; refer to Fig. 4f) by considering its narrow gap. By letting *n_R_*_1_, *n_R_*_2_, and *n_R_*_3_ be the number of moles in fluidic resistance *R*_1_, *R*_2_, and *R*_3_, respectively, the state vector **Y** of the entire system is defined as:

$$\mathbf{Y}=\left[ \begin{matrix} \begin{matrix} n_{1} & n_{R1} & n_{2} \end{matrix} & \begin{matrix} n_{R2} & n_{3} & n_{R3} \end{matrix} \end{matrix} \right]^{\mathbf{T}}$$

The magnitude of fluidic resistance *R_i_* (*i* = 1, 2, 3) with a circular cross-section is obtained as *R_i_* = 128*μL_i_*/π*d_i_*^4^, where *μ* is dynamic viscosity, *L_i_* is the length of channel (i.e., equivalent to *R_L_*_1_ and *R_L_*_2_ in the main text), and *d_i_* is the inner diameter (i.e., 1 mm in this work) ^[32]^. The molar concentration (mol/m^3^) in a i^th^ fluidic resistance is assumed to be the average of node i and node i+1:

$$\frac{1}{2}\left( \frac{P_{i}+P_{\mathrm{atm}}}{RT}+\frac{P_{i+1}+P_{\mathrm{atm}}}{RT} \right)$$

Since the gas flow rate in i^th^ fluidic resistance can be obtained by dividing the pressure difference by *R_i_*; namely, (*P_i_* – *P_i_*_+1_)/*R_i_*, the rate of change of *n_Ri_* with respect to time is calculated by multiplying its gas flow rate by the corresponding molar concentration:

$$\dot{n}_{Ri}=\frac{1}{2}\left( \frac{P_{i}-P_{i+1}}{R_{i}} \right)\left( \frac{P_{i}+P_{\mathrm{atm}}}{RT}+\frac{P_{i+1}+P_{\mathrm{atm}}}{RT} \right)$$

Note that when *i* = 3, *P*_4_ is considered zero, as the gauge pressure drops to zero across the edible valve. By assuming a constant CO_2_ gas generation rate *d*_in_ from the edible pneumatic battery (refer to Fig. 2e), the time derivative of **Y** is explicitly written as below:

$$\dot{\mathbf{Y}}=\left[ \begin{matrix} \begin{matrix} \dot{n}_{1} \\ \dot{n}_{R1} \\ \dot{n}_{2} \end{matrix} \\ \begin{matrix} \dot{n}_{R2} \\ \dot{n}_{3} \\ \dot{n}_{R3} \end{matrix} \end{matrix} \right]=\left[ \begin{matrix} \begin{matrix} d_{\mathrm{in}}-\dot{n}_{R1} \\ \left( P_{1}-P_{2} \right)\left( P_{1}+P_{2}+{2P}_{\mathrm{atm}} \right)/\left( 2RTR_{1} \right) \\ \dot{n}_{R1}-\dot{n}_{R2} \end{matrix} \\ \begin{matrix} \left( P_{2}-P_{3} \right)\left( P_{2}+P_{3}+{2P}_{\mathrm{atm}} \right)/\left( 2RTR_{2} \right) \\ \dot{n}_{R2}-\dot{n}_{R3} \\ P_{3}\left( P_{3}+{2P}_{\mathrm{atm}} \right)/\left( 2RTR_{3} \right) \end{matrix} \end{matrix} \right]$$

In our model, the edible valve is assumed to have an intrinsic resistance of *R*_3_ when *P*_3_ is higher than its threshold *P_c_* (refer to Fig. 3e-f), while the intrinsic resistance is assumed to be increased to *R*_3_×10^4^ when *P*_3_ is lower than *P_c_*_, low_ (= *P_c_* – *ΔP_v_*; refer to Fig. 4h). Due to the wide variance of *P_c_* in manufacturing of an edible valve (refer to Fig. 3e-f), *R*_3_, *P_c_*, and *P_c_*_, low_ are empirically tuned when simulating the 9 cases studied in Fig. 4. The system of state equation **Y** is solved with an *ode15s* function in Matlab.

**Supplementary Note 3**

Gelatin is used as a main material in this work due to following considerations.

**Edibility:** Other edible hydrogels can replace gelatin in terms of edibility; for example, starch, carrageenan, pectin, alginate, and agar. However, all these hydrogels will eventually undergo performance degradation over time, just like gelatin, due to moisture loss, which is a common problem of using hydrogels in the development of robotic structures. Moreover, gelatin alternatives are typically composed of multiple mixtures of these hydrogels ^[36]^, which complicates the fine-tuning of desired mechanical properties and nutritional profiles.

**Thermo-reversibility:** Gelatin typically starts melting below 40°C, and this property enables the assembly of gelatin-based components by simply applying heated gelatin liquid as a glue. Additionally, it makes the gelatin-based components easy to recycle by simply melting them with some extra water, followed by moulding them into different shapes. This also helps to save a significant amount of material cost during the prototyping stage. Instead, starch and alginate-based hydrogels are thermally irreversible and thus assembly and recyclability are limited.

**Ability to form a thin and transparent structure:** Gelatin has been widely used to create thin and almost transparent capsules in the food and pharmaceutical industries to contain other ingredients or medication ^[36]^. Similarly, here we use gelatin for manufacturing thin and semitransparent exteriors of the pneumatic battery to observe the chemical reaction.

**Exceptional melt-in-mouth quality:** The low melting temperature of gelatin (< 40°C) also enables intense flavor release when chewed, which is difficult to replicate with other edible hydrogels ^[36]^. This melt-in-mouth quality is particularly beneficial in the food sector to release a burst of palatable taste during consumption and makes gelatin easier to engineer to deliver various taste profiles depending on the intended consumers.

**Wide range of tailorable gel strength:** Gelatin hydrogels exhibit a combination of superior mechanical properties, elasticity, as well as versatility in the food industry compared to other edible hydrogels [36 – 38]. Gelatin also offers a wide range of tailorable gel strength by tuning mixing ratios, adding edible chemical crosslinkers, or utilizing the Hofmeister effect ^[38]^.

**Shell-snapping:** Our previous work showed that gelatin hydrogel can be used for producing fast shell-snapping motion by leveraging its elastic properties ^[29]^. This shell-snapping behavior is leveraged by the edible valve to repetitively release gas. Moreover, gelatin hydrogel is the only edible material that is known to be mechanically strong and elastic enough for shell snapping ^[29]^.

But using gelatin also comes with some costs and trade-offs. First, gelatin is an animal-sourced material, which could raise issues for some consumers. Also, the low melting point of gelatin prevents its use in an excessively warm environment. In addition, the mechanical properties of gelatin exhibit noticeable batch-to-batch variability even when sourced from the same manufacturer, and depend on pig breeds, feeding and rearing conditions, and material processing methods ^[39, 40]^.

**Supplementary Note 4**

The energy density of the edible pneumatic battery is evaluated as follows. A theoretically possible maximum pressure-volume work (*W*_1-2_) when CO_2_ gas is released at an absolute pressure of *P*_1_ into the environment with atmospheric pressure *P*_2_ = 101.325 kPa is:

$$W_{1-2}=nRT\ln\frac{P_{2}}{P_{1}}$$

where *n* is the number of CO_2_ gas in moles, *R* is the universal gas constant (8.314 J·mol^-1^·K^-1^), and *T* is the environmental temperature (room temperature; 298.15 K) ^[10, 23]^. Here, the case of using a 3.0 M concentration of citric acid is considered, as this exhibited the highest energy density. Additionally, *n* is obtained from Fig. 2a by converting the released CO_2_ gas mass during the chemical reaction into moles, by considering the CO_2_ molar mass (44.01 g/mol). The CO_2_ gas release pressure (*P*_1_ =86 kPa) was estimated by experimentally measuring the maximum pressure that an edible pneumatic battery can hold before breakage. Once *W*_1–2_ is calculated for each size of *D_O_*, it is divided by the gross mass of the edible pneumatic battery; the sum of chemical reagents (citric acid and sodium bicarbonate), empty citric acid capsule (23 g), and empty edible pneumatic battery case (40 g). The calculated energy density (in Joule per gram) with 3.0 M citric acid is: 1.39 (*D_O_* = 0.5 mm), 1.79 (*D_O_* = 0.7 mm), 2.22 (*D_O_* = 0.9 mm), 1.97 (*D_O_* = 1.3 mm), and 2.34 (*D_O_* = 1.5 mm).

**Supplementary Table S1**

Capillary pressure (*P_γ_*, unit: Pa) around the orifice (diameter: *D_O_*) of the citric acid capsule, where CAC refers to the concentration of citric acid liquid.

| *D_O_* CAC | **3.0 M** | **2.0 M** | **1.5 M** | **1.0 M** |
| --- | --- | --- | --- | --- |
| **1.5 mm** | 59.7304 | 60.7610 | 61.3539 | 62.4118 |
| **1.3 mm** | 68.9197 | 70.1089 | 70.7929 | 72.0137 |
| **0.9 mm** | 99.5507 | 101.2684 | 102.2564 | 104.0197 |
| **0.7 mm** | 127.9937 | 130.2022 | 131.4725 | 133.7396 |
| **0.5 mm** | 179.1912 | 182.2831 | 184.0616 | 187.2355 |

**Supplementary Table S2**

Hydrostatic pressure (*P_s_*, unit: Pa) at the orifice of the citric acid capsule with three different sizes (large, medium, small), where CAC refers to the concentration of citric acid liquid.

| Size CAC | **3.0 M** | **2.0 M** | **1.5 M** | **1.0 M** |
| --- | --- | --- | --- | --- |
| **Large** | 324.6435 | 298.6351 | 286.1578 | 273.1789 |
| **Medium** | 258.9569 | 238.2109 | 228.2582 | 217.9053 |
| **Small** | 176.8486 | 162.6806 | 155.8836 | 148.8134 |

**Supplementary Table S3**

The material mixing ratios of gelatin hydrogel (in mass) used in this study. Refer to Fabrication and Method for more details.

| **Gelatin : Glycerol : Water** | **Function** |
| --- | --- |
| 1 : 3 : 3 | Edible pneumatic actuator |
| 1 : 2.5 : 2.5 | Edible valve |
| 1 : 2 : 2 | Edible valve |
| 1 : 1 : 3 | Edible tube |
| 1 : 0 : 3 | Cylindrical housing for edible pneumatic battery and valve  Gelatin reinforcement when connecting two edible tubes |
| 1 : 0.35 : 3 | Top cover of the edible pneumatic battery |

**Supplementary Reference**

[S1] A. M. Badawy, T. A. A. O. Ganat, In: T. S. Santra, F.-G. Tseng (eds), *Rock Properties and Reservoir Engineering: A Practical View*, Petroleum Engineering, **2022**.

[S2] M. Żarska, M. Dzida, A. Apelblat, *J. Mol. Liq.* **2017**, 228, 91.
